# Using Internet of Things to Reduce Office Workers’ Sedentary Behavior: Intervention Development Applying the Behavior Change Wheel and Human-Centered Design Approach

**DOI:** 10.2196/17914

**Published:** 2020-07-29

**Authors:** Yitong Huang, Steve Benford, Dominic Price, Roma Patel, Benqian Li, Alex Ivanov, Holly Blake

**Affiliations:** 1 School of Media and Communication Shanghai Jiaotong University Shanghai China; 2 Institute of Intelligent Communication Shanghai Jiaotong University Shanghai China; 3 School of Computer Science University of Nottingham Nottingham United Kingdom; 4 Makers of Imaginary Worlds Ltd Nottingham United Kingdom; 5 School of Health Sciences University of Nottingham Nottingham United Kingdom; 6 Nottingham Biomedical Research Centre National Institute for Health Research Nottingham United Kingdom

**Keywords:** sedentary behavior, workplace, just-in-time adaptive intervention, internet of things

## Abstract

**Background:**

Sedentary behavior (SB) is associated with various adverse health outcomes. The prevalence of prolonged sitting at work among office workers makes a case for SB interventions to target this setting and population. Everyday mundane objects with embedded microelectronics and ubiquitous computing represent a novel mode of delivering health behavior change interventions enabled by internet of things (IoTs). However, little is known about how to develop interventions involving IoT technologies.

**Objective:**

This paper reports the design and development of an IoT-enabled SB intervention targeting office workers.

**Methods:**

The process was guided by the behavior change wheel (BCW), a systematic framework for theory-informed and evidence-based development of behavior change interventions, complemented by the human-centered design (HCD) approach. Intervention design was shaped by findings from a diary-probed interview study (n=20), a stakeholder design workshop (n=8), and a series of theoretical mapping and collaborative technical design activities.

**Results:**

The resulting intervention named *WorkMyWay* targets a reduction in office workers’ prolonged stationary behaviors at work and an increase in regular breaks by modifying behavioral determinants in 11 theoretical domains with 17 behavior change techniques. The delivery technology consists of a wearable activity tracker, a light-emitting diode reminder device attached to a vessel (ie, water bottle or cup), and a companion Android app connected to both devices over Bluetooth. The delivery plan consists of a 2-week baseline assessment, a 30-min face-to-face action planning session, and 6-week self-directed use of the delivery technology.

**Conclusions:**

This is the first study to demonstrate that it is possible to develop a complex IoT-enabled intervention by applying a combination of the BCW and HCD approaches. The next step is to assess the feasibility of *WorkMyWay* prior to testing intervention efficacy in a full-scale trial. The intervention mapping table that links individual intervention components with hypothesized mechanisms of action can serve as the basis for testing and clarifying theory-based mechanisms of action in future studies on *WorkMyWay*.

## Introduction

### Background

Sedentary behavior (SB) is “any waking behavior characterized by an energy expenditure of less than 1.5 metabolic equivalents (METs) while in a sitting, reclining, or lying posture” [1]. SB has negative and independent impacts on cardiometabolic health [2-6], and the health risks increase with prolonged bouts of sitting (ie, over 30 or even 60 min) [6,7]. Office workers often sit for long periods at work [8-11], which makes a strong case for SB interventions to target this setting and population [8].

The past two decades have seen an increasing number of digital technologies with various form factors (eg, personal computers, tablets, smartphones, wearables, service robots, and internet of things [IoT]) entering people’s everyday lives; they are increasingly utilized to deliver digital behavior change interventions (DBCIs) for health [9,10]. However, DBCIs in the medical and health sciences literature mostly use screen-based multimedia. Attempts to explore emerging digital interfaces beyond screens for health have been sporadic yet encouraging. These include IoT-enabled persuasive designs that overlay or embed digital information on or in the physical environment to influence behaviors at both conscious [11-14] and unconscious [15-19] levels.

Our previous work [20] has systematically scoped DBCIs aimed at reducing office workers’ SB and found that technological designs combining passive data collection with automated tailored feedback and scheduled prompts and involving several connected devices are promising for delivering just-in-time adaptive interventions. Positive user experiences are reported in studies where feedback and prompts are delivered with aesthetic and ambient media interfaces (eg, decorative objects and ambient light) that are seamlessly integrated with the physical environment and actuated over wireless connections based on real-time behavioral sensing [13,14]. However, very few of those novel modes of delivery emerging from the engineering and design fields have moved forward to the “evaluation phase” under the Medical Research Council’s framework for developing and evaluating complex interventions [21].

A potential reason could be that the development of these IoT-based interventions rarely follows systematic theory-driven intervention design approaches, such as the intervention mapping approach [22] and the behavior change wheel (BCW) [23]. This makes it difficult to develop a theoretical understanding of the mechanisms of action underlying interventions, which, in turn, prevents novel interventions and emerging research from being utilized by the wider community of behavior scientists and health intervention researchers [21].

Another possible reason concerns the complexity of IoT systems and the additional challenges they pose for intervention designers compared with developing more traditional DBCIs that are web-based or app-based. For instance, in IoT development, requirements will need to be specified for not only software, but also hardware (ie, electronics) and industrial design (ie, the casing of electronics and integration with everyday objects). Moreover, as an IoT system usually involves multiple connected devices and interfaces that work in tandem, a key challenge lies in deciding what behavior change contents and functionalities should be delivered by each of the devices as part of an integrative intervention for optimal user experience, social validity, and behavior change outcomes in workplace settings. Design decisions as such are rarely documented but are highly valuable to inform and encourage future developments of high-quality interventions using similar technologies.

In view of the above, we develop and report an IoT-enabled SB intervention named *WorkMyWay*, which is systematically grounded in theories using the BCW and balanced with stakeholder requirements for acceptability in workplace contexts. In what follows, we first introduce the methodological frameworks that have guided or inspired our design process. We then detail a five-stage development process including the methods and outcomes of each stage that have fed into the final intervention design, followed by a description of the final intervention using the Template for Intervention Description and Replication (TIDieR). In the end, we reflect on both the intervention we developed and the development process to draw implications for future research.

### Systematic Application of Theories to Intervention Development

More extensive use of theory in behavior change interventions is found to be associated with an increased effect size [9]. However, there are 83 theories of behavior change [24], many of which have overlapping constructs [25]. Therefore, the real challenge lies in selecting the most relevant theories in a systematic manner [26]. The BCW [23], developed by Michie and colleagues to support such processes, was therefore adopted to guide this research.

At the center of the BCW is the “COM-B” model of behavior, which breaks down behavioral determinants into three dimensions, with two subcomponents in each dimension, namely *capability* (psychological and physical), *opportunity* (physical and social), and *motivation* (automatic and reflective). It should be noted that the COM-B model is not a theory per se, but an abstraction of many theories about human cognition and behaviors, including the dual process model [27,28], modern habit theories [29], implementation intention [30], etc. The COM-B model can be expanded with the theoretical domain framework (TDF), which extracts 128 key theoretical constructs from 33 behavior change theories and organizes them into 14 theoretical domains [31]. The compatibility with TDF adds to the theoretical rigor of the BCW, as the TDF has been widely used and validated in studies identifying determinants of behavior change [32,33]. Using matrices from the BCW guide, intervention designers can translate the COM-B and TDF diagnosis into intervention options specified in terms of behavior change techniques (BCTs), which are considered the irreducible “active ingredients” within any behavior change intervention [34].

### Complementing Theories With a Human-Centered Design Approach

Intervention design is about not only theoretical soundness, but also appropriateness and relevance to the local context [23]. Hence, it is important to involve users (eg, office workers in the context of workplace health interventions) and other stakeholders (eg, managers of office workers) early in the design process and shape the design to their needs [35]. Moreover, the development, deployment, and upgrade of an IoT-based system are presumably more complex and therefore more costly than software systems, so it would be undoubtedly risky to exhaust a project’s resources to implement an IoT delivery system without a thorough understanding of the context of use and stakeholders’ preferences beforehand. Nonetheless, it is particularly hard for IoT novices to envision possible interactions with an imaginary IoT system beyond screen-based graphic user interfaces. This means conventional formative research approaches, such as interviewing and surveying stakeholders on “what features they want,” are insufficient to elicit useful information for a potential design.

In view of this, we consulted the human centered design (HCD) methodology that originated from human-computer interaction, a field of research within the discipline of computer science concerned with designing and studying human interactions with computing systems by drawing on methods and theories from a range of other disciplines including psychology, sociology, and design. The HCD can be a valuable complement to the BCW-guided process, with its rich repertoire of “quick-and-dirty” design methods and tools for getting stakeholder inputs before developing a fully functional product [36].

## Methods

### Overview of the Design and Development Process

**Figure 1:**
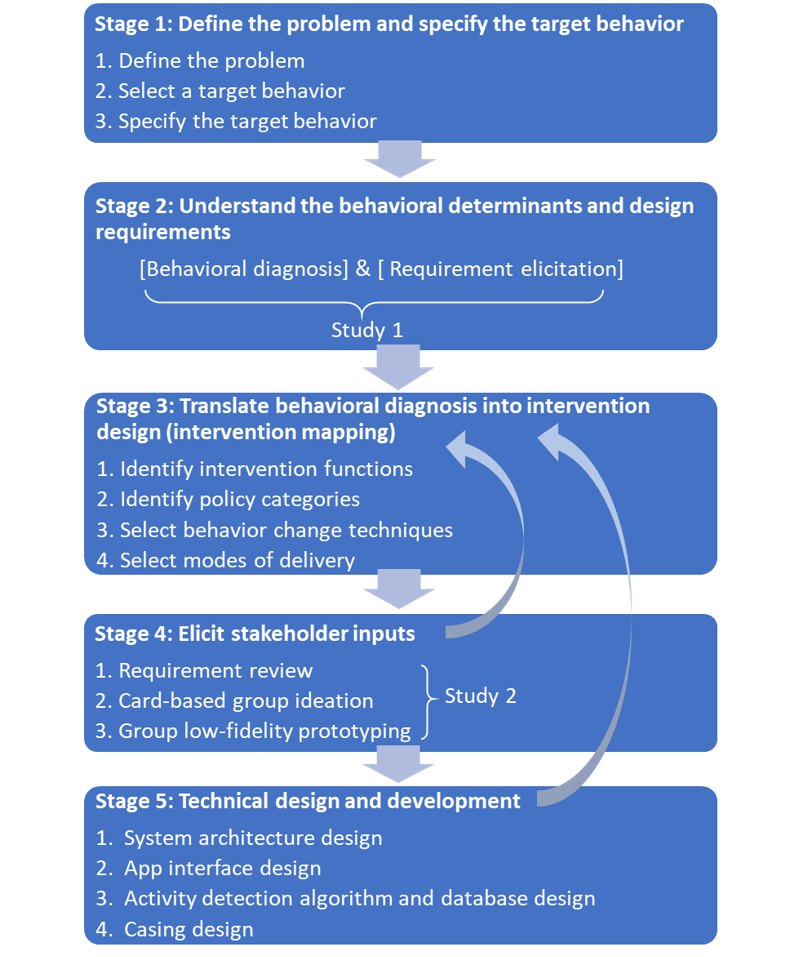
WorkMyWay intervention and technology development process.

We report how we brought together these two approaches in *WorkMyWay* development, which included five stages (Figure 1) that were iterative, meaning outputs from each stage could be used to refine the outcome(s) of the previous stage(s) whenever needed. Both empirical studies (stage 2 and stage 4) were approved by the ethics committee at the School of Computer Science, University of Nottingham (January 14, 2016, and July 06, 2016).

### Stage 1: Define the Problem and Specify the Target Behavior

Stage 1 involved three BCW-guided analytic and decision steps. Step 1 entailed defining the problem in behavioral terms and listing all behaviors that might influence the behavior of our interest. Step 2 involved selecting a target behavior from the list of candidate behaviors based on the following four criteria: (1) the likely impact of the behavior change; (2) ease of change; (3) possible spillover effects on other related behaviors; and (4) ease of measurement. Step 3 required specification of the target behavior in terms of “what, who, where, when, how often, and with whom.”

**Figure 2:**
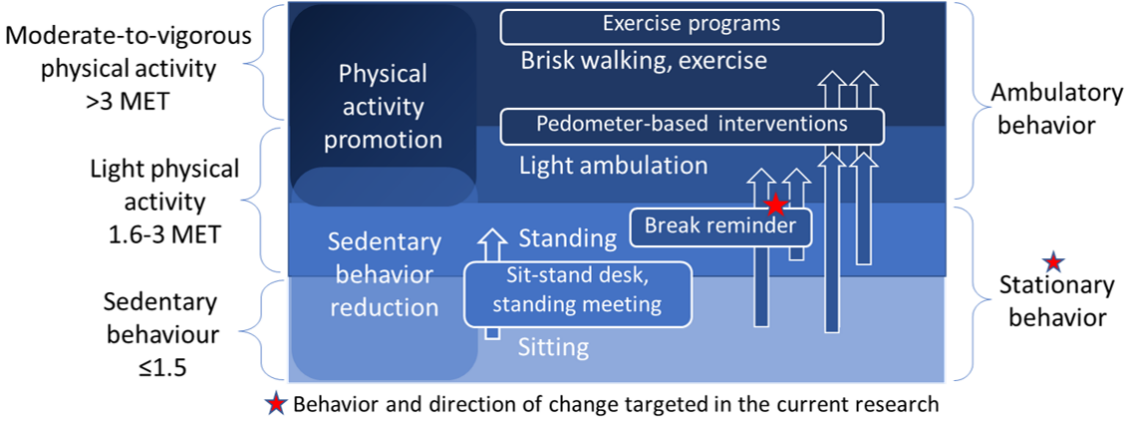
The intervention continuum from sedentary behavior reduction to physical activity promotion.

As shown in Figure 2, many published interventions have targeted physical activity (PA), although SB, on the lower end of the activity continuum, is increasingly targeted as a separate behavior in interventional studies. Targeting SB can be more effective in reducing sitting [37,38], but can have the undesirable spillover effect of increasing prolonged standing and limited promise for a positive spillover effect on ambulatory time [39-41]. Therefore, a decision was made to target prolonged stationary behavior (a combination of sitting and standing [1]) by encouraging regular ambulatory breaks, a behavior with proven cardiometabolic benefits [42]. There is yet no consensus regarding the optimal interval and duration of breaks in sedentary work. Expert advice and interventions variably promoted targets that ranged from “a 2- to 3-min light physical activity (LPA) break every 30 min of sitting” [43] to “a 5-min LPA break every 60 min of sitting” [44]. Hence, we decided to support customizable target setting with our intervention.

### Stage 2: Understand the Behavioral Determinants and User Requirements (Study 1)

As per the BCW, the next stage would entail a “behavioral diagnosis” using the COM-B and TDF to identify a full range of factors and processes requiring modification to achieve the desired behavior change. From an HCD perspective, we considered it equally important to understand the context of use and the needs of users prior to design, a step known as “requirement elicitation.” Semistructured interviews and diary keeping are both common requirement elicitation methods, with the latter particularly useful for gaining a picture of how a future system can support the user in the context of everyday operations [36]. Hence, we integrated them with behavioral diagnostic interviews commonly used in intervention design.

#### Methods

We recruited 20 participants (12 female participants) via a mailing list and posters from the University of Nottingham and two local nongovernment organizations. The participants were self-identified sedentary office workers employed in diverse office roles including admin, communication, project management, filmmaking, research, and education.

We asked each participant to keep a diary for 2 workdays and interviewed them afterwards with diaries as probes. Interviews were audio recorded with consent. The diary template (Multimedia Appendix 1) allowed participants to record details of each break experience, including the trigger, timing, location, social context, activities, and experience of the break; working tasks prior to the break; and preferences on receiving a break reminder in that moment. Participants were told that a break was defined as “any interruption in sitting” in this study. To gather design inspirations from participants’ perspectives for objects where electronics could be potentially embedded to make smart objects, one of the dairy items asked the participant to imagine potential interactions with an anthropomorphic everyday object by completing the sentence “*when… (context)…, I would like my …(object)….to say to me...(content of message)…*” Participants could also take and email photos to the researcher to illustrate the physical contexts of their breaks and the objects involved. Interview questions were constructed partly according to the COM-B and TDF to diagnose behavioral barriers and facilitators and partly around suggestions for a potential IoT delivery system (the full interview questioning route is presented in Multimedia Appendix 1).

All interviews were transcribed verbatim. Framework analysis [45] was applied to interview data for behavioral diagnosis under the COM-B and TDF, and thematic analysis [46] was applied for identifying design requirements. Content analysis was applied to diary data to identify common routines, physical environments, and objects that had triggered breaks and that could be potentially redesigned to prompt more breaks.

#### Results

Interviews ranged from 42 to 66 min. The behavioral diagnostic result from the study has been reported elsewhere [47] and hence will only be briefly summarized here. The following five COM-B components (with theoretical constructs specified in brackets) were identified as important determinants of office workers’ SB: psychological capability (eg, knowledge, cognitive resources, and skills required for monitoring and regulating break patterns), automatic motivation (eg, prolonged sitting habit and effects or emotions associated with breaks), reflective motivation (eg, beliefs about the consequences of taking breaks, perceived behavioral control, the priority and accessibility of health-related goals at work, and the intention to break up sitting regularly), physical opportunity (eg, job demands, time pressure, and organizational climate), and social opportunity (eg, social norm of prolonged sitting versus regular breaks and direct social interactions that prompt or hinder breaks). This step laid the foundation for identifying and clarifying the theoretical underpinnings for the resulting intervention.

As for the context of use, based on 291 break-related diary entries, the most common reasons that prompted participants to stand up were work-related (eg, walk between meetings and printing) (n=84), followed by the need to refill cups or water bottles (n=63), go to the toilet (n=53), do chores (eg, wash dishes after lunch and deliver envelopes) (n=48), and eat or snack (n=25). Accordingly, vessels like cups and water bottles appeared to be the objects most frequently seen in break activities. 

Thematic analysis of interview data and object messages suggested by participants in diary entries further revealed user requirements for the proposed intervention delivery system, which are summarized in Textbox 1.

**User requirements elicited from study 1.**

**Reminders**

- To be triggered when prolonged sitting is detected
- Timer should be automatically reset after I take a break
- Can be manually disabled in certain situations
- Should allow personalized settings
- Should not lock up the screen or enforce breaks when I want to work (ie, should let me retain control and autonomy)

**Feedback**

- Should provide visual feedback on my break pattern and support historical comparison and personalized goal setting
- Could provide social comparison with others (though the motivational value could vary across individuals and may encourage some workers to sit for even longer)

**Manner of communication**

- Perseverant yet flexible: the system should allow me to “snooze” it several times
- Factual and informational: the system should show me the sitting time
- Readily accessible but nonintrusive: feedback should always be there, but I can choose when to view it
- Credible and authoritative (so that it helps me justify my breaks)
- Difference between participants on preferred tone of voice: gentle and soft (eg, “maybe you wanna take a break”) versus forceful, telling off, and guilt-inducing (eg, “get up, lazy”)
- Difference between participants on preferred characteristics of the object: functional, utilitarian, and nongimmicky (eg, “just a beep or light would suffice”) versus caring, cute, or other anthropomorphic characters

**Modalities of communication**

- Tactile and visual prompts would be widely acceptable
- Audible prompts would be most noticeable but unacceptable in shared offices

### Stage 3: Translate Research Insights Into Intervention Design

The next stage entailed translating research findings into intervention design by making four major decisions on the following: (1) the broad categories of means by which an intervention changed behaviors (ie, intervention functions), (2) the policy categories used by authorities to support and enact the interventions, (3) the BCTs that best served the interventions functions, and (4) the mode(s) of delivery for implementing the intervention contents.

The BCW [23] detailed links between the TDF domains and the potentially effective intervention functions, policy categories, and BCTs. However, as the previous step identified 11 out of 14 TDF domains as relevant to the office workers’ SB, we had a whole range of nine intervention functions as potentially effective options. We then assessed the appropriateness of each option by applying the APEASE (affordability, practicability, (cost-) effectiveness, acceptability, side-effects or safety, and equity) criteria [23]. From there, we excluded “coercion” (creating an expectation of punishment or cost), as it conflicted with the user requirement for agency, autonomy, and control over work break rhythms. We also excluded “restriction” (using rules to alter the opportunity to engage in the target or competing behavior), as it was impractical and unacceptable to restrict office workers’ access to seated workstations or prevent them from going to long-seated meetings.

We discussed the seven policy categories (ie, service provision, guidelines, fiscal measures, communication or marketing, environmental or social planning, regulation, and legislation) with human resource managers and staff wellbeing leads in several organizations. It was concluded that only service provision was relevant at the point of intervention design before firm evidence of effectiveness was established.

The process of selecting BCTs is detailed in Multimedia Appendix 2, with examples of mapping shown in Table 1. In a nutshell, we first adapted the matrix from the BCW guide [23] to map the relationships between intervention functions and the TDF-based behavioral diagnosis; we then identified potentially effective BCTs for each cell in the matrix based on the BCW guide [23]. This resulted in a range of BCTs selected for each cell with illustrative intervention contents. We then removed some BCTs (eg, restructuring the social or physical environment) that did not meet the APEASE criteria.

We set out to utilize novel modes of delivery, particularly IoT-enabled smart objects and wearables, as much as possible. However, considering the upfront development cost (the “affordability” criteria in APEASE) for digitizing the delivery of all BCTs and the high likelihood of making changes to the intervention design after the feasibility and piloting phase (“cost-effectiveness” criteria in APEASE), we decided to build a minimum viable IoT product with all the essential technological functions and complement it with face-to-face sessions and email communication for BCTs that required complex dialogue support and individualization (eg, goal setting and action planning). Examples of intervention mapping following the BCW are presented in Table 1 (see Multimedia Appendix 2 for full details).

**Table 1:** Examples of intervention mapping following the behavior change wheel.

Constructs/mechanisms of action targeted	Intervention function	BCTs^a^	Intervention components and *modes of delivery*
Memory, cognitive overload, and behavioral regulation	Enablement	Conserve mental resources, feedback on behaviors, and self-monitoring	Use *wearable* trackers to automatically monitor sitting time, and an *app* provides daily feedback to enable user to self-monitor day-to-day changes in break patterns.
Belief about capabilities	Education	Feedback on behaviors	An *app* presents daily summary of and feedback on the sit-break pattern.
Prospective memory, cognitive overload, and goal priming	Environmental restructuring	Conserve mental resources, prompts and cues, and add objects to the environment	Add or augment *objects* that facilitate the performance of breaks; use the *object* to cue breaks naturally associated with the object (eg, augment a cup to cue tea breaks).
Breaking habit, self-efficacy, and implementation intention (goal accessibility)	Enablement	Action planning	*Researcher* guides the person to set up plans to combat prolonged sitting by specifying the frequency and duration of breaks, including developing “if-then” rules that use an *IoT object* as the cue.
Habits and contingencies	Training	Habit formation	*Researcher* guides the person to develop automatic responses to the introduced *stimuli (the IoT object)* through repetition.

^a^BCT: behavior change technique.

### Stage 4: Elicit Stakeholder Inputs (Study 2)

In this stage, we invited stakeholders (eg, managers and occupational health consultants working for organizations) to review both theory-based and user-centered design requirements generated from the previous stages in order to assess potential acceptability of the proposed design in various organizational contexts from managerial or experts’ perspectives. We decided to conduct a design workshop, which is a common HCD method for bringing together a cross-section of stakeholders to not only identify issues that need to be addressed (similar to focus groups), but also produce design solutions collaboratively [36]. Card-based ideations and low-fidelity (lo-fi) prototyping activities are frequently employed in design workshops. The former usually relies on ideation decks created for specific design briefs with contents illustrating parameters directly relevant to the design problems of interest to prompt group creativity and discussions [48]. Lo-fi prototyping refers to creating noninteractive mock-ups of potential design solutions with little or no programming or engineering effort, which are useful to represent system requirements and to allow the design team and stakeholders to evaluate candidate solutions and identify problems with them before investments in technical development [36].

#### Methods

Our workshop was funded as an industry engagement activity by the Balance Network [49] and promoted by workplace health specialist networks and word of mouth. Many work health specialists expressed interest to learn about outcomes from the workshop, but only eight could make it on the workday when it was hosted. The eight delegates (two female and six male) represented large organizations, small-to-medium–size enterprises, and public and private sectors, and all had an interest in enhancing employee health and wellbeing.

Materials for the workshop are provided in Multimedia Appendix 3. The half-day workshop started with requirement review, where each participant completed an individual worksheet that asked them to rate the COM-B and TDF behavioral determinants elicited from Study 1 in terms of *“to what extent does this statement reflects what you have observed in your workplace?”* and *“how important do you think this factor is in determining micro-break behaviors?”* We then presented the participants with a diagram illustrating the proposed intervention delivery system with the following three components: a wearable activity tracker, a smartphone app, and an IoT cup or water bottle (without delineating interactions or user interfaces in detail). We brought some commercially available IoT cups (eg, Cuptime, Moikit Ltd) and passed them around to give participants a more concrete idea of how embedded sensing, data processing, wireless connectivity, and different digital interfaces could fit together and what features such products were capable of delivering. Participants were encouraged to challenge the proposed system design and to raise potential deployment issues in workplace settings. We also encouraged participants to suggest alternative objects as the medium for delivery. 

Thereafter, participants split into two groups to ideate system features by completing group worksheets. The process was supported by a deck of 25 persuasive IoT ideation cards that we specially designed for this project, which consisted of three categories of “opportunity” cards, namely physical, social, and sensing opportunities. The contents were created based on inspiration from previous IoT decks [50], persuasive design frameworks [51], and the BCT taxonomy [34].

After the ideation, participants were introduced to the concept of lo-fi prototyping. In addition to common lo-fi prototyping materials like paper and Play-Doh, we supplied our participants with *LittleBits* (LittleBits Electronics Inc), an educational kit with modular electronics that could snap together with small magnets to make circuits attachable to Lego cups. This innovative combination of tools and materials was intended to help participants envision and evaluate various modalities (eg, light, vibration, and audio) through which they could be reminded by a potential smart cup in the workplace. The prototyping activity was concluded with a showcase session, where each group reported back on their design ideas and prototypes, with the other group asking questions and suggesting improvements to the design.

#### Results

The requirement review did not particularly challenge behavioral insights from Study 1; participants were receptive to the proposed intervention and technology, and raised questions mostly concerning technical feasibility (eg, *“can the system detect a break if the user doesn’t not take the cup with him/her during the break?”*).

**Figure 3:**
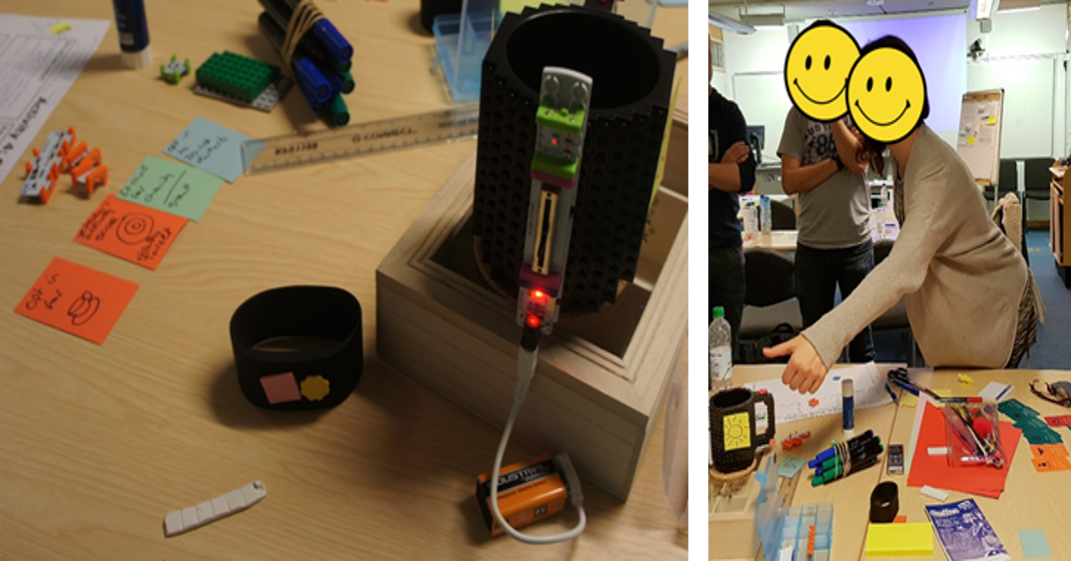
Design idea generated by Group 1.

Group 1’s design (Figure 3) validated the requirements for personalization and user autonomy elicited from Study 1 and revealed new requirements concerning specifics about the interaction flow and feedback interfaces. They prototyped a cup with an ambient display that could be personalized to individual users. They suggested the display, for instance, could gradually reveal a picture of the user’s dog to suggest that the user was increasingly in need of a break (represented by the light-emitting diode [LED] dimmer switch in the prototype in Figure 3). The proposed design also included two buttons for the cup; one to allow the user to temporarily disable all reminders during meetings and the other to “snooze” reminders (the user could postpone a visual reminder for breaks for up to three times, after which a vibratory reminder would be triggered on one’s wristband). Group 1 also created paper prototypes for a companion app, which illustrated features like automated tailored feedback and rewards for regular breaks. Finally, Group 1 preferred that all data be kept private online, although users could choose to share their app screens with friends and co-workers offline to foster competition. Group 2’s design revealed more nuances in the balance between harnessing social influences and respecting individual privacy. The design was targeted at open plan offices with the culture of co-workers taking turns to bring back drinks from the kitchen for others so that most people could work for longer without interruptions. Group 2 decided to harness this existing culture by identifying and prompting the most sedentary person in the office to stand up for a break. In this way, each user would be motivated to break up sitting more proactively to prevent one’s own cup from buzzing and embarrassing oneself in the office. In addition, Group 2 suggested fostering social cooperation and team competition by deploying, in the staff common room, a dashboard displaying each office’s aggregate activity data. Nonetheless, Group 2 emphasized that the dashboard should not give away any behavioral data identifiable to individuals.

### Stage 5: Technical Design and Development

A range of tools, including diagrams, wireframes, mockups, and pseudocodes, were used to document the requirements throughout the previous stages. These were then compiled into a requirement specification document (Multimedia Appendix 4), which was frequently referred to and refined over the process of technical implementation.

It was decided at this stage that BCTs related to social influences (eg, social support, social comparison, and demonstration of the behavior by managers and workplace champions) would not be technically implemented for the following three reasons: (1) both Study 1 and Study 2 suggested that the technology would likely trigger conversations and competitions between office mates offline, even without explicit instructions for social comparisons in the app; (2) the integration of social functions would complicate the architecture design and increase development time; (3) both studies suggested potential ethical controversies associated with sharing data about an individual’s break patterns in terms of surveillance on employees’ work behavior and that it might impel some workers to sit for longer in an attempt to impress others.

#### Development Platform, System Architecture, and Application Programming Interfaces

We researched ways to digitally augment an everyday vessel and enable it to track the physical footprints of itself and its owner, and to deliver meaningful just-in-time adaptive interventions. After an audit and comparison of different IoT development platforms, we selected the *MetaWear* RG (MbientLab Inc) platform, which includes an accelerometer, a color LED, and a Bluetooth Low-Energy (BLE) module on-board, as well as an Android software development kit (SDK). The SDK was important as it allowed us to focus on software development (eg, streaming accelerometer data captured by the wearable device over a BLE connection in real-time) without much investment in hardware engineering. A diagram that illustrates the system architecture and application programming interfaces used is included in Multimedia Appendix 4.

#### Interface Design

Based on Android guides for user interface and navigation design [52], we created wireframes to illustrate the information architecture (eg, layout and navigation) and interaction flow for the app (Multimedia Appendix 4). Tabs were chosen for lateral navigation between three sibling sections, namely “track,” “history,” and “rewards,” which were expected to be used most frequently. The infrequently used and discrete options (“about,” “user setting,” and “researcher setting”) were accessible from a drop-down menu at the top right corner.

#### Algorithm and Database Design

Previous accelerometry-based activity classification algorithms predominantly applied thresholds to processed accelerometer output in the form of counts per epoch (CPE), which was indicative of activity intensity [53]. Owing to a lack of established cutoff points between SB and LPA for the MetaWear RG sensor, we conducted a series of structured data collection sessions, in which the wearer undertook various activities (eg, sitting while typing, sitting while writing, sitting while talking with hand gestures, sitting while doing torso twist, standing up, walking for five steps, and walking continuously) and identified the cutoff point between SB and LPA breaks based on visual inspection. The data revealed that sitting activities featured a CPE of 5 or less most of the time and 5-10 occasionally (eg, torso wrist), continuous walking featured a CPE of 23-30, and standing activities that involved mild ambulation (eg, standing up to open the window blinds and fetching a file in the same room) had an CPE of 10-25. We decided that the system should pick up two types of events that we wanted to consider and encourage as “breaks,” including a burst of high-intensity movement (CPE >25) that signifies walking to a different room and a continued period (>20 epochs or 5 min) of mild ambulation (CPE >10) that potentially involves doing chores in the room.

**Figure 4:**
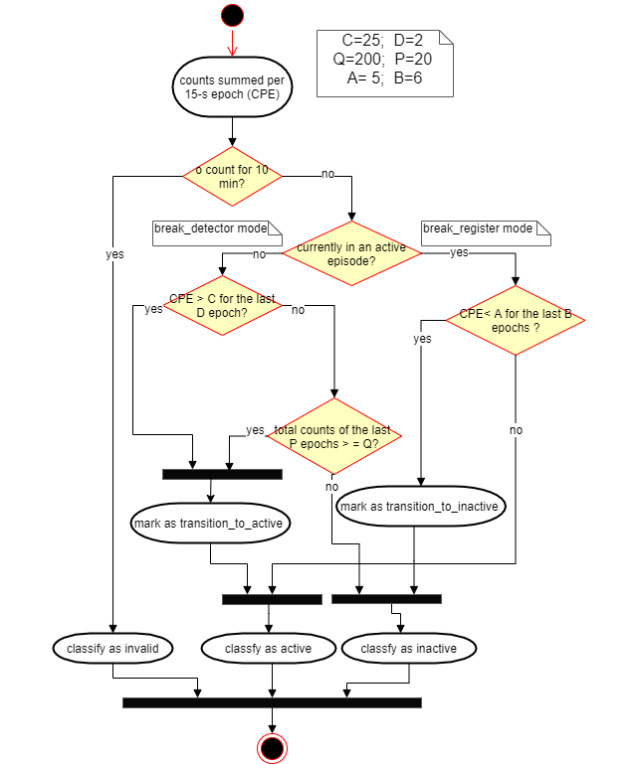
Activity classification algorithm.

From there, we developed an algorithm that differentiated inactivity (ie, SB and standing still) and activity (ie, ambulatory breaks) based on a combination of activity intensity (ie, CPE cutoff points; parameters C, Q, and A in Figure 4) and temporality (ie, number of continuous epochs with CPE exceeding the cutoff points; parameters D, P, and B). Moreover, the algorithm featured a break detection mode and a break register mode to reduce frequent transitions between SB and breaks that were most likely caused by sporadic hand movements (eg, fidget and gesture).

To support tailoring, we included a password-access menu in the app that allowed the researcher to change the default parameters to adjust the sensitivity of the break detector for individual participants if required. For instance, the value of C could be raised by up to 5 CPE if the participant had particularly vigorous wrist movements in a sitting position that caused false positive break detections and the value of D could be lowered to 1 CPE if the break facility was close to the participant’s office and if the participant preferred a quick trip to the break facility to be recognized as a break. The researcher could lower the value of P to 1 CPE, which would make the system not recognize mild movements as breaks at all.

Finally, while designing the algorithm, we considered the research needs for assessing fidelity and quantity of delivery for the *WorkMyWay* intervention. Hence, we designed the algorithm to detect an invalid tracking period caused by nonwear or technology failure based on the number of continuous epochs of zero counts. Moreover, we requested that the system log usage of the tracking and goal setting functions and record the timestamps of prompt delivery. In this way, fidelity could be operationalized as the percentage of tracking time that is valid and the percentage of prompts successfully delivered, and adherence to different functions could be operationalized as the number of days of use of each of the functions.

#### Casing Design

We discussed internally and consulted with product designers on several options to fix the electronics to the vessel, and we considered the ease of use for participants, as well as the ease and cost of production. We decided to three-dimensionally print cases for the *MetaWear* electronics using a template from the *MetaWear* manufacturer and make the printed case attachable to any vessel with a belt or Velcro tape. This would make the limited sets of *MetaWear* electronics reusable (ie, could be taken off and stuck to another vessel for the next participant without hygiene problems) and easy to handle for both researchers and users (ie, could be removed from the cup or bottle during meetings, for charging, and at the end of the study).

## Results

### Final WorkMyWay Intervention

We describe the resulting *WorkMyWay* intervention. To increase the quality of reporting, a TIDIeR checklist [54] is presented in Multimedia Appendix 5. Intervention materials are presented in Multimedia Appendix 6. The text below briefly describes the IoT-enabled delivery system and intervention protocol. 

### The Technological Delivery System

**Figure 5:**
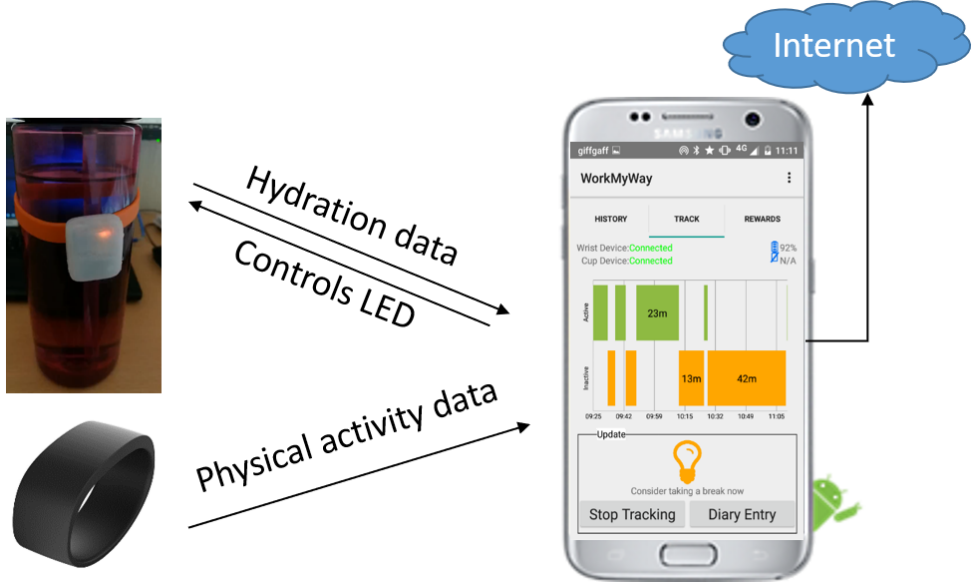
The resulting intervention delivery system. LED: light-emitting diode.

**Figure 6:**
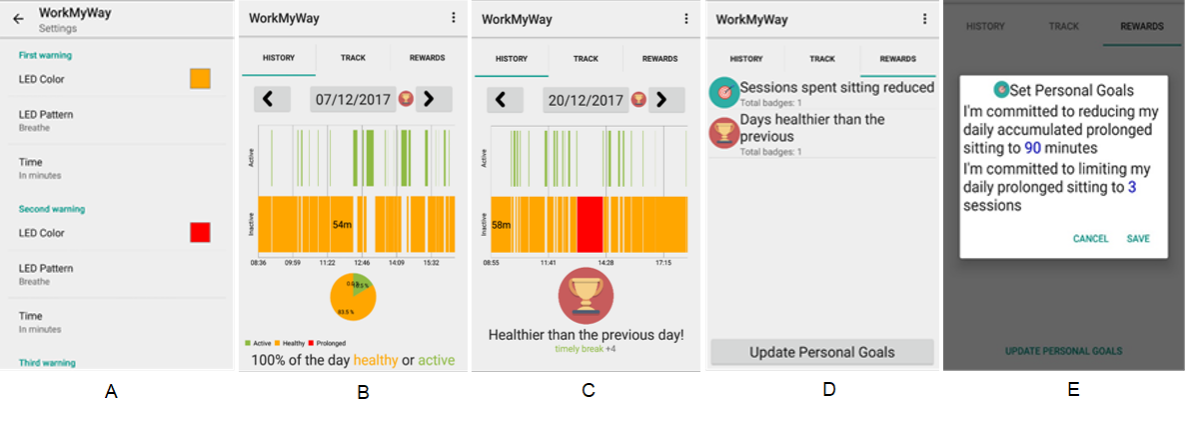
Screenshots of the WorkMyWay app.

The resulting intervention delivery system consists of a PA monitor (called “wrist device”) to be worn by the user, an LED reminder to be attachable to any cup or bottle (called “cup device”) of the user’s choice, and an Android app connected with both devices over Bluetooth (Figure 5). The wrist device automatically tracks movement and constantly syncs data with the app. The app differentiates activity and inactivity with the algorithm described above and actuates the LED according to the user’s period of inactivity and prespecified rules (Figure 6A). At the end of each day, the app provides more detailed visual and numeric summaries of daily SB (Figure 6B) and rewards behavioral improvements and goal achievements with trophies and badges (Figure 6C). The “reward” section in the app allows the user to review previous rewards (Figure 6D) and adjust goals (Figure 6E). There is an “about” page with scientific facts about SB and information about the study, which is accessible from the drop-down menu. In addition, a *WorkMyWay* Lite app is made for baseline measurement, which works with the wrist device alone and merely provides “tracking” functionality (ie, no feedback).

### Intervention Delivery Protocol

**Figure 7:**
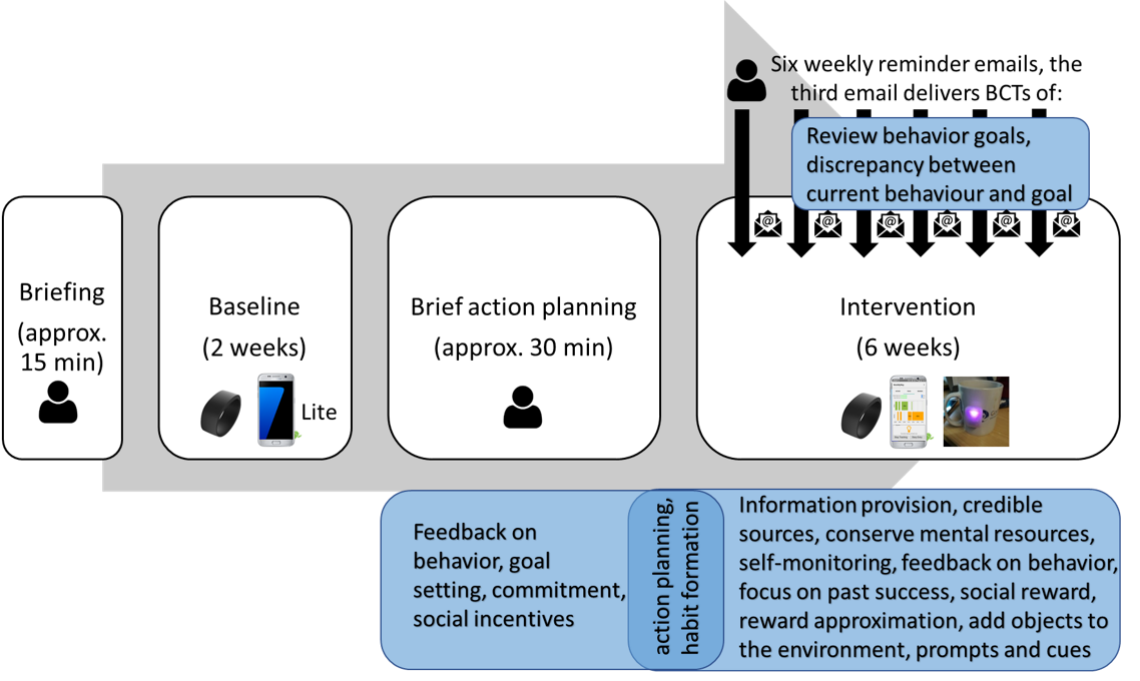
The timing and dosage of delivery for each intervention component and the underlying behavior change techniques (BCTs). The underlying BCTs are specified in blue boxes, and the modes of delivery are indicated by icons (eg, wrist device, smartphone app, cup device, and researcher’s email).

The timing and dosage of delivery for different intervention components are summarized in Figure 7.

First, to set up personalized goals, the intervention requires information about individual participant’s baseline SB, which needs to be collected with the *WorkMyWay* Lite system for 2 weeks. A trained researcher or occupational health consultant should help the participant set up the connection and give instructions on technology use at a face-to-face briefing session. A one-page two-sided “study cheat” sheet is also provided to each participant (Multimedia Appendix 6). After the 2-week baseline period, the researcher revisits the participant, replaces the Lite app with the full *WorkMyWay* app, fixes the “cup device” to a vessel of the participant’s choice, and delivers a 30-min brief action planning (BAP) session to prepare the participant for the upcoming 6-week intervention period. BAP is a support technique that mirrors the spirits of motivational interview [55] and is aimed to facilitate participants in forming action plans that they feel confident to achieve. For instance, instead of prescribing a desired end state for the participant, the researcher can ask, *“now that we’ve looked at your baseline data and talked about prolonged sitting and health, is there anything you would like to do for your own health in the workplace in the next week or two?”* The participant will be guided to make specific action plans in terms of how often they would like to break up sitting in different contexts and to make use of the three configurable LED events to support execution of the action plans. The full BAP protocol is detailed in Multimedia Appendix 6.

After the BAP session, the participants will use the full *WorkMyWay* app with the wrist and cup devices during their own office hours, with minimum intervention from the researcher for 6 weeks. To enhance adherence, a weekly reminder email is sent to the participants by the researcher on each Monday morning. The third weekly reminder email contains an extra message that completes the BAP protocol by prompting the participants to review their “history” in the app, compare performance against the goals initially set by them at the BAP session, and adjust the goals and reminder settings in the app if necessary. The email also asks the participants if they want to have the break detection sensitivity threshold adjusted to suit their individual work contexts and preferences.

After 6 weeks, the researcher contacts each participant to schedule a debriefing interview, where subjective experiences of the behavior change intervention and technology use can be discussed.

The researcher or practitioner needs to be trained in both troubleshooting the *WorkMyWay* system and applying the BAP techniques. All sessions need to take place at the participant’s workplace.

## Discussion

### Principal Results

This paper describes the development process, as well as the final design of a novel SB intervention delivered with an IoT system. We have followed a systematic and comprehensive theory-driven development approach while drawing upon HCD methods to involve stakeholders in the design process. The resulting intervention draws upon a total of 17 BCTs to target behavioral determinants in 11 theoretical domains. We reflect on the novel design of our delivery system, as well as the development process to discuss lessons for future research.

Our *WorkMyWay* system is complex, combining the following three distinct types of devices that deliver different intervention components to target different behavioral determinants:

1. A screen-based component (the app) that focuses on delivering the intervention functions of education, persuasion, and incentivization. This approach has been extensively used and tested in existing DBCI research [56].
2. A wearable component that focuses on delivering the intervention function of enablement by automatically collecting and livestreaming PA data over a Bluetooth connection, which essentially offloads the human cognitive task of self-monitoring to the technology. This is also an established approach in both academic literature and commercial products [57].
3. An IoT component (the digitally augmented vessel) that is intended to deliver the intervention function of environmental restructuring and enablement, as it is seamlessly integrated into an office worker’s working environment and daily routine to create awareness of sitting time with subtle prompts and cues for breaks. This is the novelty of our approach.

A key lesson from our technology design is therefore, first of all, to recognize the different modalities of communication afforded by different devices and carefully choose suitable media to deliver intervention contents with the appropriate level of obtrusiveness for different moments. In our case, the types of contents to be delivered range from a detailed timeline of activity episodes for end-of-day reflection, through “at-a-glance” environmental cues for in-situ awareness and actionable information, to unobtrusive and unnoticed data capture. Our various interfaces embody a range of interaction styles that operate in the foreground (ie, occurs in the center of attention) versus background (ie, attentional peripheral) [58], which has been debated substantially in human-computer interaction. The advocacy for designing background interactions dates back to Mark Weiser’s vision [59] that technology will weave into everyday life and become unnoticed eventually, which was followed on by the call for calm computing [60]. Our design of the glanceable IoT component mirrors the ethos of calm computing by making it stay in the periphery of the user’s attention most of the time without intruding on the user and only move to the center of attention when prolonged sitting is detected and just-in-time intervention is required. On the contrary, our app is designed to counter balance this embeddedness and calmness with the need to engage, stimulate, and provoke users to be reflective at the end of each workday [61]. This also highlights the importance of the intervention protocol that specifies when and how each technological component is to be used and for how long (ie, the required dosage of each component).

Furthermore, we argue that IoT-enabled objects should not be seen as a replacement for screen-based apps and wearables, but rather as an addition. Those smart objects are particularly suitable for delivering certain intervention functions from the BCW, such as enablement and environmental restructuring, whereas screen-based media are better reserved for the intervention functions of education and training. Hence, instead of designing a whole IoT intervention delivery system anew, designers might consider a more incremental approach, extending existing apps and wearables with complementary IoT objects that will eventually contribute to a more complete ecology of DBCI technologies.

Another aspect we want to reflect on is the design of data processing, which has implications for requirements like personalization, user autonomy, and privacy. Given sufficient training data, we recognize the potential to replace our “quick and dirty” algorithm with a more sophisticated machine learning algorithm that can learn about and adapt to individual patterns of behavior. However, it should be noted that our manually designed classification rule has the advantage of transparency, so that researchers and clinicians delivering the intervention can potentially adapt it for individuals by directly tweaking its various parameters, which is easier than adjusting a “black box” machine learning algorithm. We also noted the need for sharing data between the various technological components. This requires attention to reliable networking and a potentially encrypted data transmission protocol that is a hidden but often difficult aspect of technical development, with important implications for data security, privacy, and ownership. In the context of PA tracking in the workplace, who has access to data is a nontrivial issue and needs to be handled with caution to minimize the chance of employer surveillance or peer pressure that can sabotage the health initiative.

### Strengths

A major strength of this study was the application of the BCW and related frameworks (eg, TDF, the BCT taxonomy, and TIDIeR) to structure the development and description of the intervention, which helped clarify theoretical underpinnings, active ingredients, and the final design. There has been a call for more thorough reporting of intervention design and development processes [62], and papers documenting the design and development of DBCIs with those frameworks have emerged over the past few years [63-65]. However, these papers tended to report on the design and development of interventions delivered with less advanced technologies, such as web pages, smartphone apps, and SMS, rather than IoT technologies. To our knowledge, our study is the first to systematically apply all BCW steps to develop an IoT-enabled intervention and specifically reflect on this technological approach.

Another strength of this study was related to factoring in the need to assess fidelity and dosage of delivery at the time of designing the database. This meant the right type and structure of data were requested to be captured and stored to allow monitoring of user interactions with individual functions, as well as the whole intervention. The intervention mapping table (Multimedia Appendix 2) together with our monitoring data will be useful for separating and clarifying the effects of individual intervention components, which could contribute to the endeavor to establish links between BCTs and specific mechanisms of action in the field of DBCI research [66].

A third strength of the design process was the seamless integration of the bottom-up HCD approach into the top-down theory-driven intervention design process. Other studies drawing on similar approaches tended to implement BCW and HCD in distinct studies or phases [64,67]. Our approach was slightly different as we embedded HCD in BCW-guided studies. For instance, Study 1 served the dual purposes of behavioral diagnosis under the BCW framework and requirement elicitation under the HCD methodology. Study 2 could be seen as a public and patient involvement activity [68] in the context of health intervention design, though we moved beyond public and patient involvement to empower stakeholders with two innovative HCD methods. Ideation cards provide an accessible and “bite-sized” representation of design knowledge (including theory-informed BCTs and technological opportunities) for use by stakeholders from various backgrounds during collaborative design sessions. The cards essentially act as a bridging mechanism between theories and practical design solutions. Lo-fi prototyping, including the use of an IoT maker kit and loose materials, enables stakeholders to become more “hands on” in the design process and engage with emerging technologies without needing to acquire software or electronic engineering skills. We suggest that both methods can complement more traditional interviews and focus groups to elicit stakeholder requirements for novel futuristic modes of delivery (ie, smart objects) while grounding design solutions in theories.

### Limitations and Future Work

First, our final design had a rather rough look, as we used the MetaWear hardware, SDK, and three-dimensional printing template to reduce production difficulty and cost. Developing a finely finished IoT product ideally requires a team of product designers and electronic engineers, as well as software and data engineers. With that said, IoT technologies evolve so rapidly that an IoT-based intervention likely needs to be improved over time after it is developed. Hence, we compromised by building a “minimum viable product” with all the essential components for a proof-of-concept study before investing heavily in developing a finely finished product. The Medical Research Council framework also suggests a phased approach to evaluating complex interventions, starting from feasibility studies targeted at each of the uncertainties in the design and moving on to a pilot and then a definitive trial [21]. Hence, we suggest deploying and evaluating the current version of the WorkMyWay intervention in real office-based workplaces on a small scale, with focus on assessing the acceptability and feasibility of the various components and the protocol, identifying barriers and facilitators to use, and clarifying the mechanisms of action prior to pilot and full-scale randomized controlled trials.

A second drawback of this research concerns the small and self-selected sample in both studies, which limits the generalizability of the findings. Self-selection filtered out those unconcerned about the issue of SB or lacking control of break behavior at work. The wider acceptability and effectiveness of WorkMyWay will need to be demonstrated by conducting evaluative studies with more representative samples in diverse office-based workplaces.

### Conclusions

This paper documents the design and development of WorkMyWay, an IoT-enabled behavior change intervention to reduce workplace SB. The development process applied behavioral theories systematically while drawing on HCD methods. The resultant intervention, including its content, rationale, and delivery, is detailed to allow replication. Future studies are needed to evaluate the feasibility of the intervention in office-based workplaces and the efficacy of the intervention in improving office workers’ behavioral and health outcomes.

## Appendix

Multimedia Appendix 1 Study 1 materials (stage 2).

Multimedia Appendix 2 Mapping behavioral determinants to intervention components following the behavior change wheel (stage 3).

Multimedia Appendix 3 Study 2 workshop materials (stage 4).

Multimedia Appendix 4 Requirement specification document (stage 5).

Multimedia Appendix 5 Template for Intervention Description and Replication (TIDieR) checklist.

Multimedia Appendix 6 Intervention materials.

## Abbreviations

APEASE: affordability, practicability, (cost-) effectiveness, acceptability, side-effects or safety, and equity
BCT: behavior change technique
BCW: behavior change wheel
HCD: human-centered design
IoT: internet of things
LED: light-emitting diode
LPA: light physical activity
MET: metabolic equivalent
PA: physical activity
SB: sedentary behavior
TDF: theoretical domain framework
