# Supplementary material for: Using Internet of Things to Reduce Office Workers’ Sedentary Behavior: Intervention Development Applying the Behavior Change Wheel and Human-Centered Design Approach
Source: JMIR Mhealth Uhealth. 2020 Jul 29;8(7):e17914. doi: 10.2196/17914 (PMC7424484; doi:10.2196/17914)
Supplement: Multimedia Appendix 1 [file mhealth_v8i7e17914_app1.docx]

## Materials for study 1 (stage 2 of the development process)

## Diary protocol and materials (participant self-administered on paper)

For each study day, you need to

1. Use the **“Workday Episode”** form to record your workday as continuous series of **“episodes”*,** like scenes in a film. Give each episode a brief note that will help you remember it. Write down the approximate times at which each episode began and ended.
2. On top of that, for each **non-sitting episode**, you need to
3. **take a photo** that illustrates the physical contexts (tools and products used, location, environment) of each event while you are in the situation
4. complete the **“Work Break Experience Form” **** (either paper or e-version, please tick the format you have completed)

* Episode: In this study, an ***episode*** *is a continuous engagement in a certain activity with unchanged posture (e.g. sitting or non-sitting).* So you are expected to *log the start and end time of an* ***episode*** *whenever you leave and return to seat.* The episodes people identify usually last between 15 minutes and 2 hours and can take the form of an uninterrupted period of sitting, a short bout of comfort break and a longer bout of lunch break.

**Break: In this study, the term **“break”** refers to any termination in sedentary time (i.e. any activities you engage while not sitting). Even if you stand just briefly (e.g. stretch out, tidy up your desk, and talk to colleagues over the cubicle wall), it's considered a break; or even if you stand up and move about for work-related purposes (e.g. print & photocopy, collect parcel, visit another office), it's also considered a break. In the occurrence of any of the above “breaks”, you are expected to take a picture and complete a “**Work Break Experience Form”**.

#Sedentary breaks: You might have respites in your seat (e.g. checking Facebook, watching a YouTube video at desk over lunch), please report those under the question “What were you doing in the previous sitting episode?”

##Enchanted objects: imagine you are in a futuristic sci-fic world where everyday mundane objects can understand your needs and talk to you.

**Workday summary - Sample**


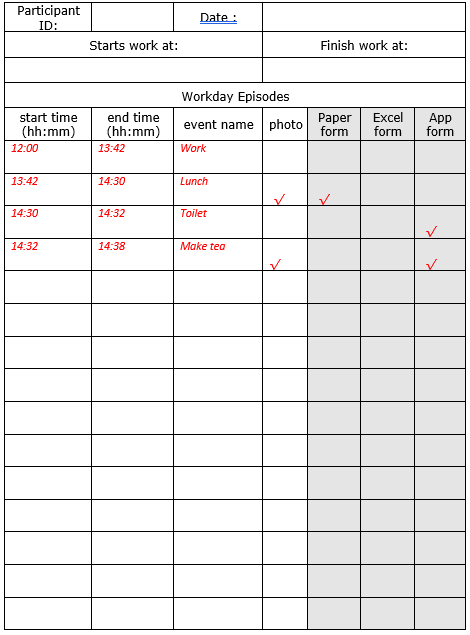


**Work Break Experience Form – Sample**


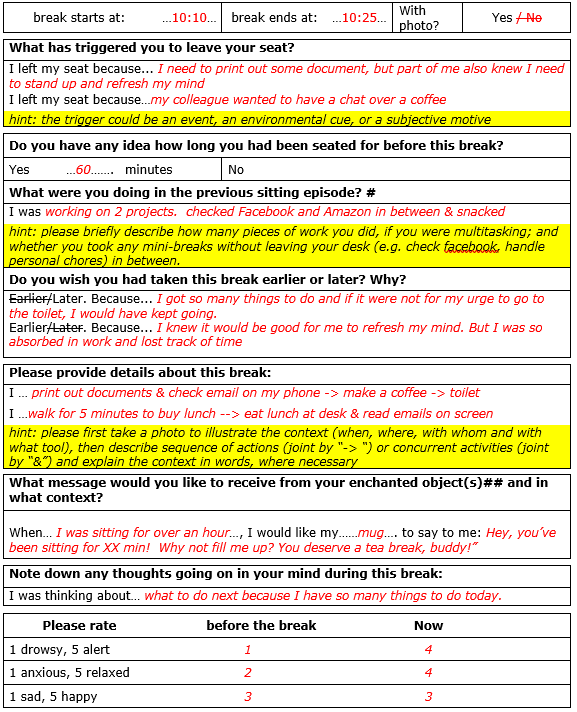


**Work Break Experience Form**


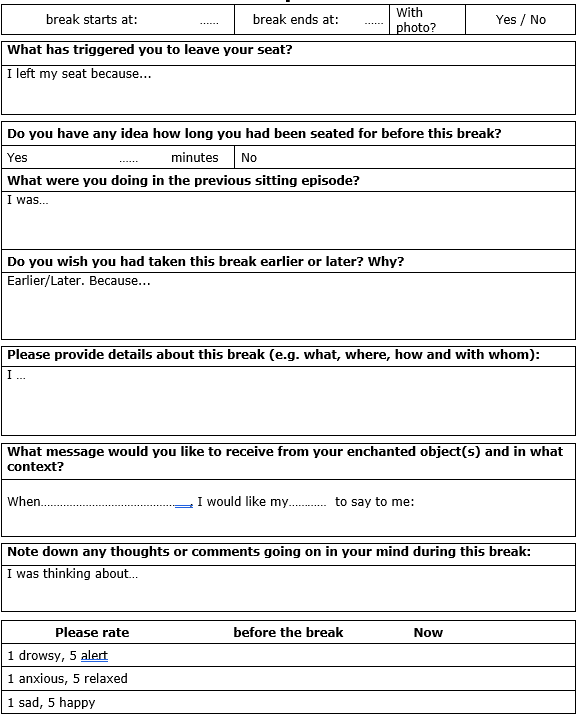


## Interview questioning route

The retrospective interview at the end of the 2-day study period will be semi-structured. Questions are likely to vary across individual respondents depending on images collected. The following questions are illustrative of the type of information to be gathered from interviews.

Theme (Heading), opening and transition questions (regular), *sub questions* (italic).

### Study experience and reflection on diary

What’s your experience of taking part in the study, apart from the object?

- *Did the action of keeping a diary and taking photos change your behaviour in any way?*
- *Did you look at your own diary materials at the end of each day? How did you feel then?*

### Discussion on occupational sitting

**Ref Mot (Beliefs about consequences/capabilities):**

**Compare two work break styles:** *given the same amount of total sitting, which of the two do you think is better? Why?*

- *What benefits do you hope or believe it Style 2 has?*
- *What harms do you think Style 1 has?*
- *How easy or difficult do you think it is to do style 2?*

Here are some graphs and statistics about your two days. Is there any surprise? What do you think? Fix data

**Ref Mot (beliefs about conse/cap, intentions, goals):**

So what do you think of your current amount of sitting everyday?

- Do you think that’s too much or just alright? What impact do you think it has?

**Psychological capability (Memory, attention, decision processes):**

- If it were not for this study, would you always remember how long you’ve been sitting? How easy or difficult do you find it to keep track of how long you’ve been sitting at work

**Psychological capability (Knowledge):** What do you think would be the ideal work break pattern for you? How certain are you about it?

What’s good about it?

1. productivity at work,
2. mood during the workday,
3. energy level at the end of the workday
4. physical health and fitness

If mention a lot about regular breaks:

- *How attractive are those benefits? / Would the benefits of this ideal work break pattern be rewarding enough for you to make a change?*
- **Intention:** *Is taking regular breaks something you generally intend to do?*

*If yes, on a scale of 1-7, how effortful do you feel it is to keep doing it?*

*When you sit for longer than 60 minutes, what prevents you from breaks?*

If mention none about breaks:

- - *are you aware of any harms of sitting for over 60 minutes at a time during work? (short- vs. long-term consequences)*
  - *Are you* *convinced that taking regular micro-breaks have significant health benefits?*

**Goal:** Have you set it as a goal to stop sitting for too long? Compared to the goal of completing your work, to what extent is taking regular breaks throughout working day a priority for you? What about keeping health in general?

- ***Psy cap (Decision/attention/memory):*** *do you tend to think about those benefits we talked about during work?*

**Self-identity:** Do you see yourself as someone who pays attention to one’s own health and fitness? Do you see yourself as a workaholic?

### Facilitators/barriers

***Objective (organisational/social)***

How difficult or easy do you think it is to find time to take a little break?

Is there any work that requires long period of concentration to get into the flow?

Are there any other factor that prevents you from taking breaks away from desk?

What’s the culture of work breaks like in your workplace?

- *How do you think your manager/supervisor perceive taking regular breaks away from desk? Are they approving or discouraging?*
- *What about colleagues? Do you feel part of a “crowd” when taking*

***Subjective strategies (psychological capability):***

**Decision/automatic mot:** Is XXX something you usually do? *Did you plan it? Or was it a random act? Or is it like a habit?*

***Behavioural regulation:*** *Do you set any rules for yourself regarding when you should stand up and move around?* Do you set any triggers to prompt yourself?

***Self-Monitoring****: Do you have system to help you monitor whether you have taken regular breaks on work days? Would you find visual feedback on your sitting and breaks, like the graph I produced, helpful?*

***Potential technologies:*** Do you have any technologies or tools that tracks your period of inactivity?

*Would you find it helpful to have a piece of technology that 1) monitors and displays your sitting time 2) send idle alert?*

***Potential interventions:*** *e.g. How would you feel about standing up and talking to your office mate? Or whenever someone comes to talk to you, you stand up?*

**Automatic motivation:**

- Habit: Would you say that generally you are in the habit of sitting for over 60 minutes/taking regular breaks? If not, what would be helpful in developing/breaking that routine/habit?
- Does taking a break evoke an emotional response?

***Break activity/contents – Automatic Motivation***

Do you have much choice over what to do during your breaks?

Can you think of a particular work break activity that can recharge your energy effectively and benefit your health?

- *Would it occur to you do to this activity when you need to take a break?*
- *Would a tool that suggests break activities be helpful to you?*
- From your images, I can see you do…. *From images, you seemed to stand up while […carrying on working on screen, paperwork (e.g. filing), talking on the phone/checking phone messages, stretching out].* Did I miss anything?

Let’s think of out the box. What other activities you would like to do during work breaks, if we forget about the physical constraints your current workplace may have? **Physical Opportunity/Automatic motivation**

What facilities do you have access to in the workplace during work breaks at the moment? What other facilities do you want to have easy access to? Physical Opportunity

Do you feel your break time **experience** itself is rewarding enough at the moment? How do you think your break time experience can be enhanced?

**Beliefs about capability:** how confident do you feel about forming a habit of taking more regular breaks and shortening your average sitting about, with the support a technology that has the features we discussed?

### Potential design

If you can design an intelligent system with any kind of objects that remind you to take breaks, what features would you like it to have?

**Approaches:**

Remind to take breaks based on idle time: How would you feel about being interrupted at work with a recommendation to take breaks from sitting?

Change content: no one asks you to take breaks; but you have a “break” manual that suggest activities to keep you more active within the breaks you usually take?

Detect fatigue: …

**Objects:**

Your XX seemed quite central in your work breaks, how often are you not around your water bottle. What about mugs? Did you use one or more mugs?

What about this - a smart water bottle with an embedded sensor that captures your sitting time and step counts, and it communicate to you about your physical activity level.

Would you find such an object pleasant or annoying? Would you keep using it?

Preferred medium:

Prefer to receive in what context?

Potential for personalisation?

What tone of voice you would like your object to speak to you in?
