# Supplementary material for: Using Internet of Things to Reduce Office Workers’ Sedentary Behavior: Intervention Development Applying the Behavior Change Wheel and Human-Centered Design Approach
Source: JMIR Mhealth Uhealth. 2020 Jul 29;8(7):e17914. doi: 10.2196/17914 (PMC7424484; doi:10.2196/17914)
Supplement: Multimedia Appendix 2 [file mhealth_v8i7e17914_app2.docx]

**Figure 1. Candidate intervention functions to target the COM-B and TDF domains based on the matrix from Michie et al. (2014) pp. 116**

*Cell 12: according to the BCW, environmental restructuring was not commonly used to target the TDF domain of goal; however, the literature suggested environmental cues could be used to heighten accessibility of goals.

Psy cap - psychological capability

Ref mot – reflection motivation

Aut mot – automatic motivation

Phy opp – physical opportunity

Soc opp – social opportunity

## Figure 2. Excluded intervention contents because of not passing the APEASE criteria

##

**Intervention mapping table (selection of BCTs were based on Michie et al. (2014) pp.151 – pp.158)**

| **Cell# in Figure 1.** | **Constructs/ Mechanisms of action targeted** | **BCTs** | **Intervention components and modes of delivery** |
| --- | --- | --- | --- |
| 1,3, 4,6,7 | Knowledge, beliefs about consequences, behavioural intention | Information about health consequences, credible source | 1. App provides recommendations on healthy break intervals with explanation of scientific rationale and emphasize that the information is from credible sources |
| 19,  20 | Retrospective memory, cognitive overload, behavioural regulation | Conserve mental resources, feedback on behaviours, self-monitoring | 2. Use wearable trackers to automatically monitors sitting time and App provides daily feedback to enable user to self-monitor day-to-day changes in break patterns. |
| 2 | Belief about capabilities | Feedback on behaviours | 3. App presents daily summary of and feedback on sit-break pattern. |
| 5,  8 | Belief about capabilities, positive/negative affect | Focus on past success | 4. The App prompts the participant, at the end of each day, to look at the App feedback on break pattern and to verbally list moments s/he has managed to take timely breaks. |
| 21,  22 | Goal (distal/proximal), beliefs about capabilities | Goal setting (behaviour), discrepancy between current behaviour and goal, review behaviour goals | 5. Researcher prompts the person to set goals (e.g. "I want to limit my prolonged sitting within 3 episodes per day) in the App and to review and adjust goals regularly with email reminders. |
| 15 | Intention | Commitment | 6. Researcher ask the person to use an “I will” statement to affirm or reaffirm a strong commitment to change the behavior. |
| 16, 17, 18 | Rewards (distal/proximal), goal (distal/proximal) reinforcement, positive affect | Social incentive, social rewards, reward approximation | 7. The researcher informs the person that the App will congratulate him/her for achieving any reduction in prolonged sitting. |
| 20, 21, 22 | Breaking habit, self-efficacy, implementation intention (goal accessibility) | Action Planning | 8. Researcher guides the person to set up plans to combat prolonged sitting by specifying the frequency and duration of breaks, including developing "if-then” rules that use an IoT object as the cue |
| 11,*12, 13, 14 | Prospective memory, cognitive overload,  *goal priming | Conserve mental resources, prompts and cues, add objects to environment | 9. Add or augment objects that facilitate the performance of breaks; use the object to cue breaks naturally associated with the object (e.g. augment a cup to cue tea breaks) |
| 9, 10 | Habits, contingencies | Habit formation | 10. Researcher guides the person to develop automatic responses to the introduced stimuli (the IoT object) through repetitions. |
| 23, 24, 25 | Social support, group conformity, organisational culture/climate | Social support (practical and general) | #11. The person could form teams and foster peer support to promote engagement with the intervention (not intentionally incorporated as part of the intervention) |
| 26-32 | Social comparison, group norm, modelling, social support, negative affect | Demonstration of the behaviour, social comparison, information about other's approval | #12. The person could see other's sitting patterns and share strategies (not implemented in *WorkMyWay* technology, but could happen spontaneously and voluntarily between co-workers offline) |
