# Supplementary material for: Using Internet of Things to Reduce Office Workers’ Sedentary Behavior: Intervention Development Applying the Behavior Change Wheel and Human-Centered Design Approach
Source: JMIR Mhealth Uhealth. 2020 Jul 29;8(7):e17914. doi: 10.2196/17914 (PMC7424484; doi:10.2196/17914)

## Study 2 (stakeholder design workshop) materials

### Individual worksheet for the design workshop with stakeholders

| **Individual Worksheet Your Name: ____________** | | | |
| --- | --- | --- | --- |
| **COM-B** | **Statements on office workers' barriers and facilitators to breaking up sitting with hourly micro-breaks in the workplace** | **To what extent does this reflect what you've observe in your workplace?**  **(1-not at all, 5-very)** | **How important do you think this factor is in determining micro-break behaviour?**  **(1-not at all, 5-very)** |
| psychological capability | Easily lose track of time when engrossed in work |  |  |
|  | Don't attend to bodily needs for breaks |  |  |
|  | No idea of the total number of breaks or episodes of prolonged sitting that have happened during a day |  |  |
|  | The decision to take a break or not needs to consider progress with the current task, physical and mental fatigue, next appointment arrangement etc. |  |  |
| Reflective Motivation | Unconvinced of health benefits of micro-breaks |  |  |
|  | Micro-breaks interrupt flow and are thus counter-productive |  |  |
|  | Concerned that other people will notice and negatively perceive break behaviours. |  |  |
|  | A technology that automatically tracks sitting, provides prompts/cues for breaks and visual feedback on my pattern would give people more confidence in improving my break pattern |  |  |
|  | Keeping a healthy work style is a low priority compared with work achievement. |  |  |
|  | Have thought about it but haven't informed a strong intention to improve work-break pattern |  |  |
|  | Taking micro-breaks is in conflict with professional standard/identity |  |  |

| **COM-B** | **Statements on office workers' barriers and facilitator to breaking up sitting with hourly micro-breaks in the workplace** | **To what extent does this reflect what you've observe in your workplace?**  **(1-not at all, 5-very)** | **How important do you think this factor is in determining micro-break behaviour?**  **(1-not at all, 5-very)** |
| --- | --- | --- | --- |
| Automatic Motivation | Have habits, routines and ingrained behavioural patterns that contribute to regular breaks. (e.g. drinking water/tea, refilling vessel regularly) |  |  |
|  | Have habits, routines, ingrained patterns and obsession-compulsion that contribute to prolonged sitting behaviours (e.g. impulse to power through) |  |  |
|  | Breaks away from seat evoke positive affect |  |  |
|  | Feel guilty for taking breaks. |  |  |
|  | Less likely to take breaks when feeling stressed. |  |  |
| Physical Opportunity | The organisation allows flexibility in how employees complete work and doesn't encourage presenteeism |  |  |
|  | Heavy workload and tight deadlines impel me to sit continuously for longer than I would love to |  |  |
|  | People have access to software/Apps/gadgets for prompting breaks (please give names in the last column) |  |  |
|  | Existing prompts/cues for breaks have limitations (please give reasons in the last column) |  |  |
| Social Opp. | Co-workers invite each other to take micro-breaks together |  |  |
|  | Spontaneous chats by the side of a break facility (e.g. coffee machine) is common |  |  |
|  | **social norm:** most co-workers are good at taking regular micro-breaks and there is no pressure on sitting down to work |  |  |

### Group worksheet for the design workshop


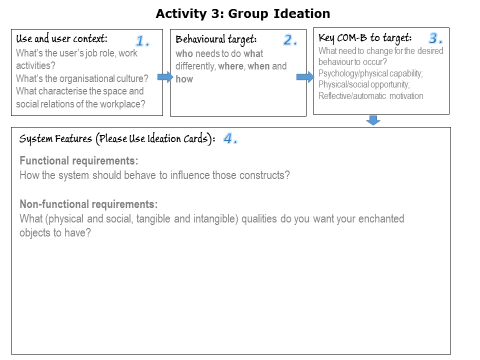


### Persuasive IoT ideation cards


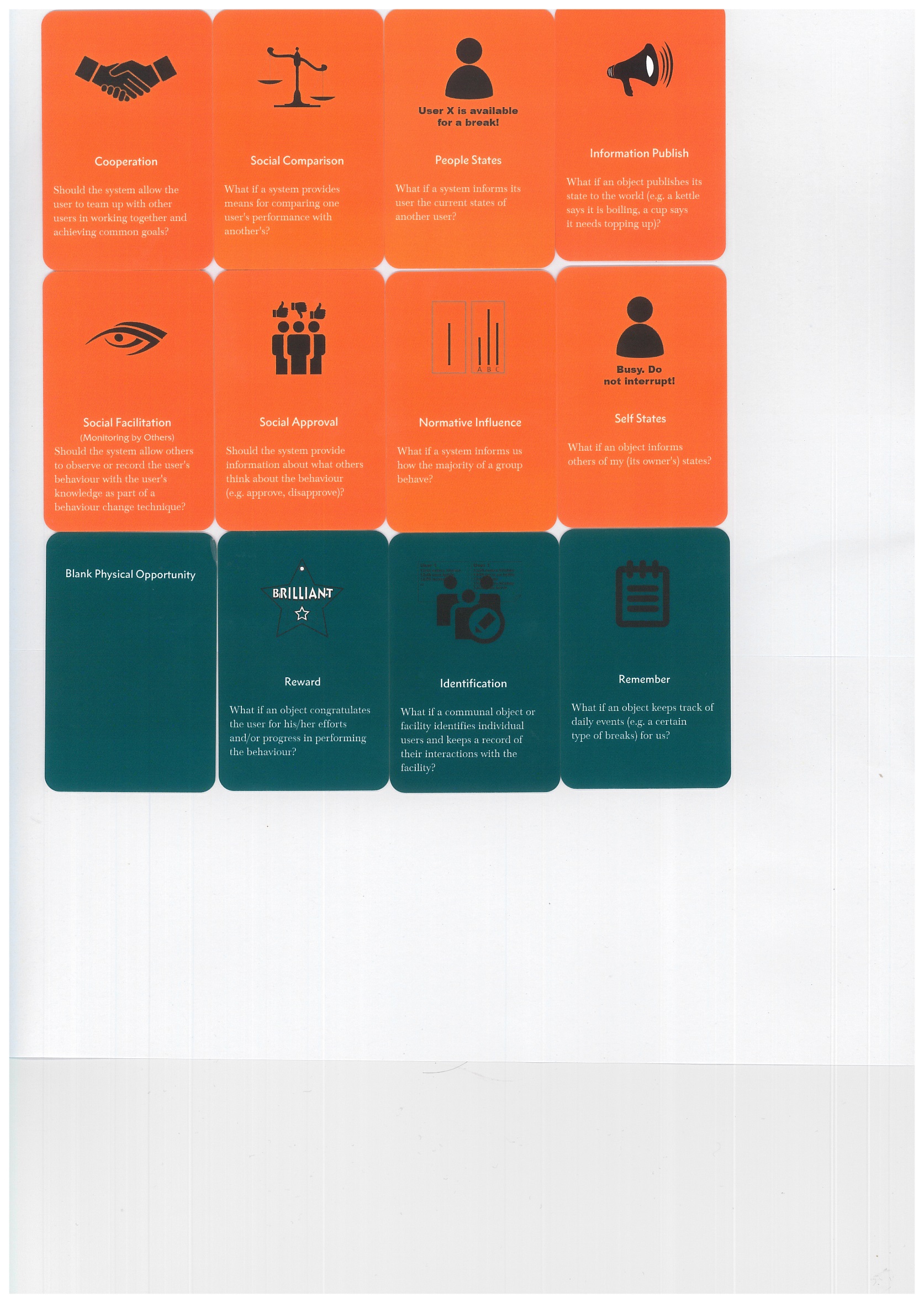


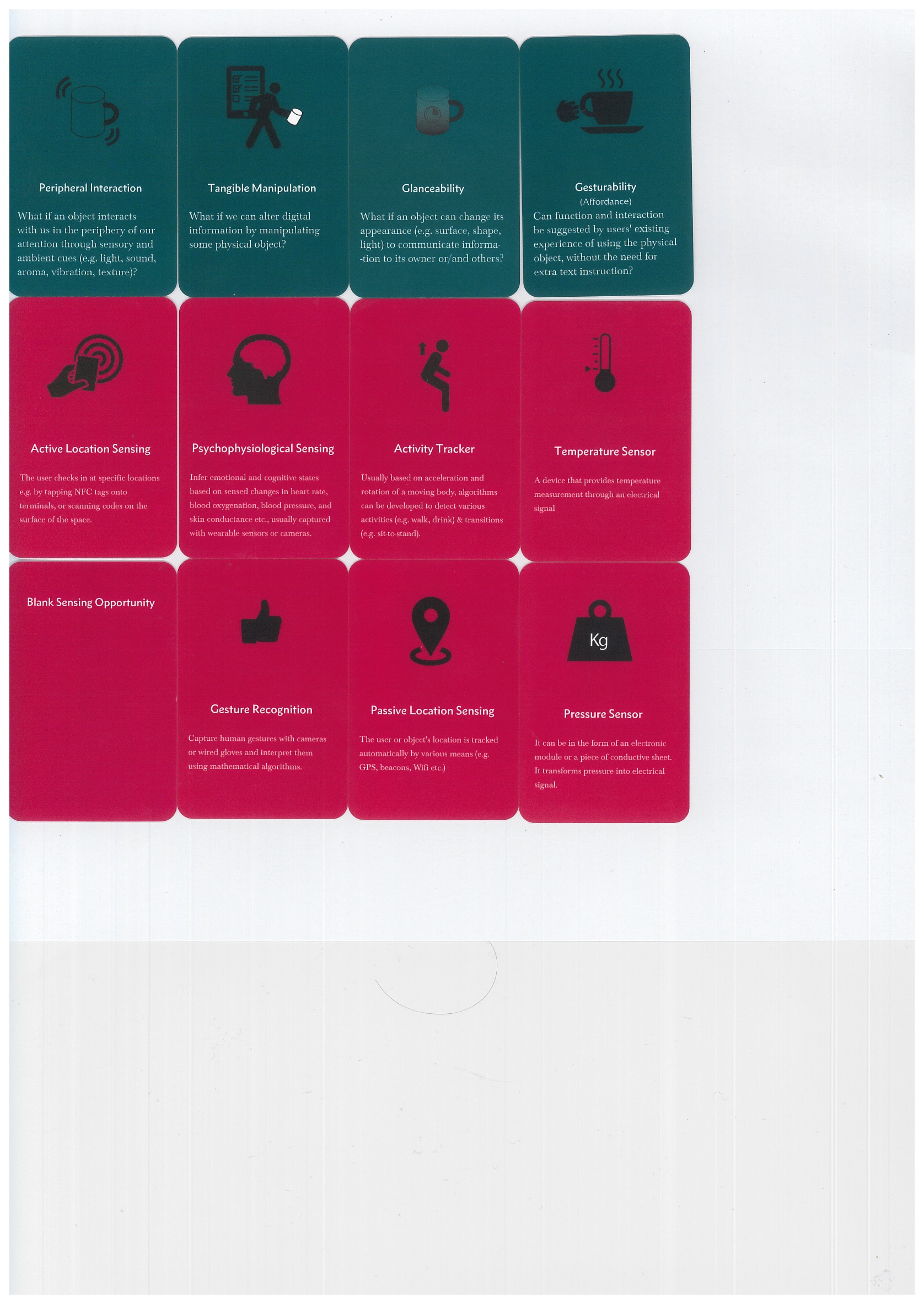


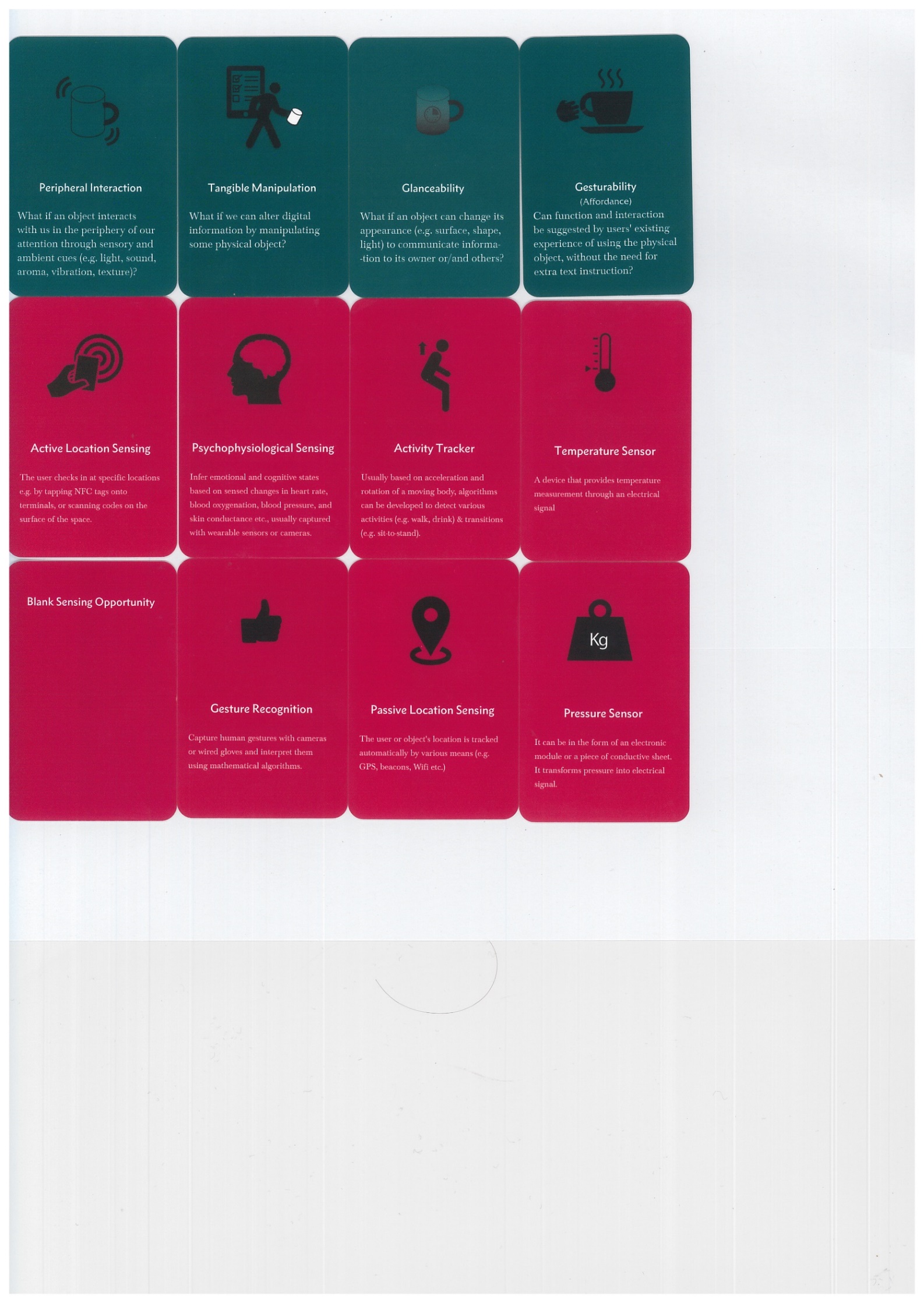


### Cards used by Group 1


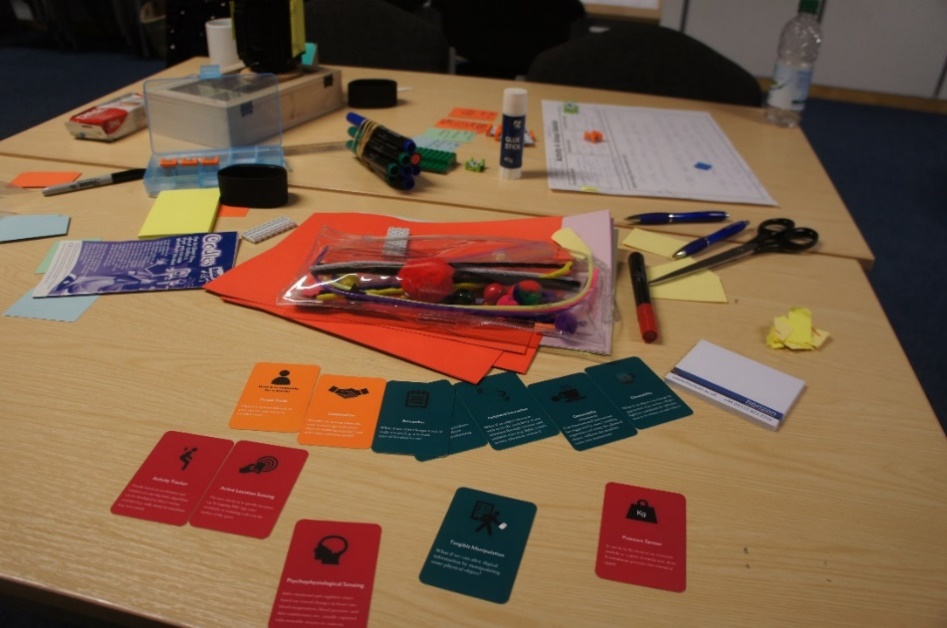


### Cards used by Group 2


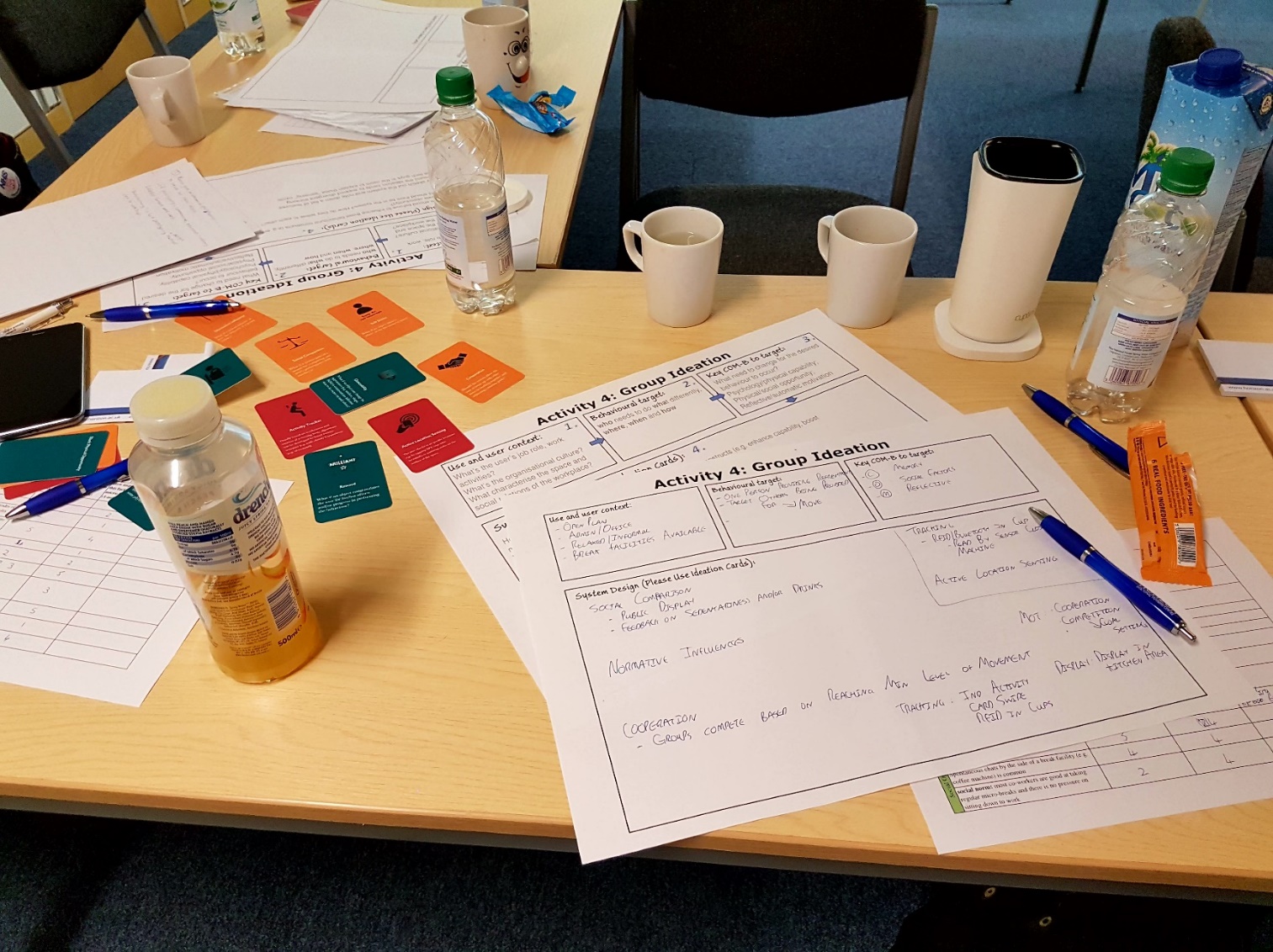

Supplement: Multimedia Appendix 3 [file mhealth_v8i7e17914_app3.docx]
