# Supplementary material for: Using Internet of Things to Reduce Office Workers’ Sedentary Behavior: Intervention Development Applying the Behavior Change Wheel and Human-Centered Design Approach
Source: JMIR Mhealth Uhealth. 2020 Jul 29;8(7):e17914. doi: 10.2196/17914 (PMC7424484; doi:10.2196/17914)
Supplement: Multimedia Appendix 4 [file mhealth_v8i7e17914_app4.docx]

**Requirement specification document**

grey shades represent those requirements not implemented in the end for technical and practical reasons

**Overview of the system**

The system will consist of 3 components – a wearable activity tracker, a mug with attached computing and an iOS (or Android) App (Figure 1). Both the wearable activity tracker and the computing part attached to the mug will be built on the *MetaWear* RG platform, which is a tiny (26mm x 17mm x 2.5mm) development board with built-in accelerometer, gyroscope, temperature sensor, BLE connectivity, rechargeable battery and (optional) vibrating motor. The platform comes with iOS/Android SDK. Details on the development board can be found here [https://store.mbientlab.com/product/*MetaWear*-rg/](https://store.mbientlab.com/product/metawear-rg/).

## System diagram


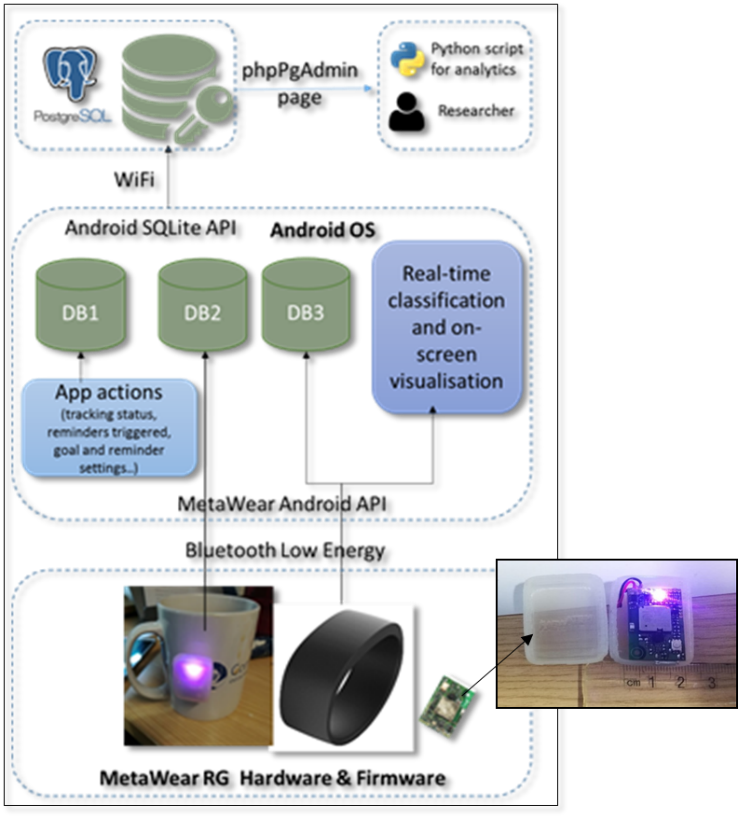


Figure 9 System architecture for *WorkMyWay*

**Core functions (implement first)**

**Interaction Functions:**

- The wearable activity tracker should keep track of the current sedentary period and constantly upload this “sedentary time” to the smartphone App via Bluetooth Low Energy (BLE).
- The *MetaWear* API/SDK has included step detection and motion event triggers on both iOS and Android (<https://mbientlab.com/iosdocs/latest/accelerometerbmi160.html#notify-when-placed-flat>) (<https://mbientlab.com/androiddocs/latest/bmi160_accelerometer.html#step-detection> ). Thus for ease of development, sedentary time can be operationalised as period of no steps in this app. So we only need to have a variable that stores the duration of the current sedentary episode (ie. time since the last step was detected); once step patterns are detected using the provided “Notify on Step” event handler, the current sedentary period should be reset to 0 min and the motion tracker should start recording step numbers. (alternative algorithm was implemented see Section 5.4.3.3)
- Some structured testing and recording may need to be carried out by the lead researcher in collaboration with the developer to validate the *MetaWear* step detection algorithm/logic.
- The App actuates the LED and vibrating motor attached to the mug via BLE based on the following rules:

1. If sedentary period ∈ (45, 55) (unit: minutes), then turn on a yellow light, representing the message that “You can consider a break now!”
2. If sedentary period ∈ [55, 59) (unit: minutes), then the light turns to green, meaning “You should take a break now!”
3. If sedentary period > = 59 min, then flash a green light, meaning “You are reaching the end of the time window to get score. Take a break now!”
4. If the reminder is on && the user tapped on the *MetaWear* board attached to the mug, the reminder will be turned off for 10 minutes (ie. “Snooze”). But snoozing won’t affect the sedentary time record or reminders scheduled in the system for the next stage; only after 3 snoozes will scheduled reminders be cancelled.
5. If during the reminder period, step patterns are detected from the Wearable *MetaWear*, the App will turn the light into a green breathing light for about 3 seconds to acknowledge that the break has been detected and that sedentary time has been reset.

- The App should feed the data back to the user in the form of an activity timeline as well as numeric summary:
  1. Daily inactive minutes: xx minutes (the sum of sitting longer than 60 min)
  2. Longest episode of inactivity: XX minutes
  3. Types of data collected and visualised will be detailed in the next section

**Data Sync and Storage:**

- When a step is notified, ideally the *MetaWear* should cache **step number** locally, in case the user didn’t take the phone with him/her, and upload to the App once connection is available again (the *MetaWear* turned out not to work like this as the manufacturer claimed). The *MetaWear* board has the storage for 10K – 15K 4byte sensor entries with timestamp, which, in theory, is more than sufficient for storing step number during a micro-break. The *MetaWear* API has provided a logging class for accessing the on-board flash memory (<https://mbientlab.com/androiddocs/latest/logging.html>).
- The App should be able to store the above behavioural data about the user for at least 12 hours locally on the phone:
  - The **start/end times and duration of each sedentary episode**; the **start/end times and duration of each break**, and **number of steps taken in each break**; “timestamped” snooze record.
- There should be a scheduled time on each day for the App to upload all data to a database server, where data will be stored over the study period (>=4 weeks). There should also be a “sync” button in the App so that the user can manually initiate synchronization any time. (it was set to automatically synced in near real time)


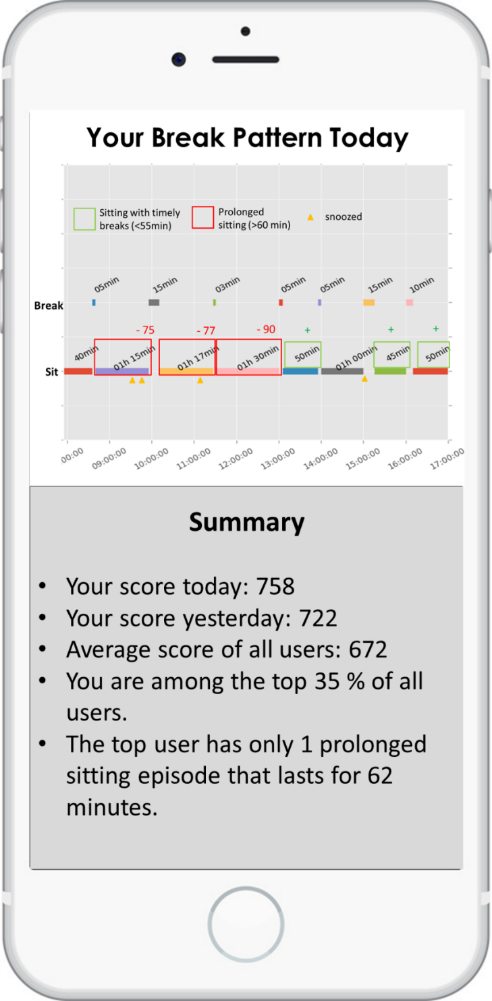


Figure 2 Example App UI for data feedback

**Research administration:**

- The researcher can access, query and download data from the server with password login.
- The researcher should be able to tweak parameters such as the sedentary time threshold for triggering the reminder, the modality of the reminder (whether vibrates or not, and choose light colour) etc.

**Add-on functions (requirements will be discussed and finalized between the researcher and the developer)**

- There can be a function for the user to set break reminder interval. But this function can be disabled and made invisible to the user by the researcher.
- The *MetaWear* board attached to the mug can capture and upload raw data about the mug’s movements to the App. An algorithm needs to be implemented in the App to determine if a movement pattern constitutes a drinking event or not, potentially by comparing the tilt angle to a threshold that is adapted to the estimated amount left in the mug. (could not implement this because of difficulty with streaming accelerometer axial data)
  - If a drink event is detected, the App will actuate a breathing blue light on the *MetaWear* attached to the mug to acknowledge that “This drinking event is recorded”.
  - If the user has not drunk from the mug for 30 minutes, the App will turn on the LED light on the mug into blue to indicate “you need to keep hydrated”.
- When the user starts walking (ie. App notified of step by the wearable board), the App should pause drink detection on the mug (to avoid mistaking steps for drinking) and trigger step detection on both boards.
- Similar to the above, step numbers captured by both boards should be cached in the on-board flash memory during the micro-break and upload to the phone when re-connected, just in case the user takes the mug but leaves the phone behind. After receiving timestamped step counts from both boards, the App should then have a function that decides if the user has taken the mug with him/her based on the similarity/difference between two step patterns. If similar, then this can be assumed as a “refill” event. Temperature sensor data may be utilized to increase the accuracy.(water break event was simply operationalized as high intensity movement of the cup device detected)
- The App records the timestamps for both of the above events and uploads such records to the database. As illustrated in Figure 3. the user can choose to make them visible on the timeline.
  - This is to demonstrate that a mug with embedded sensing can make mundane micro-breaks somehow accountable and more memorable, as it puts the wearable physical activity data into context. This has value to both the researcher who wants to understand the mechanism of behavior change and the group of users who just fancy the idea of quantified self and technology-mediated reflection. Thus this function should be configurable by the user (ie. technology probe) so as to probe into how users interpret and utilize data captured by everyday objects.


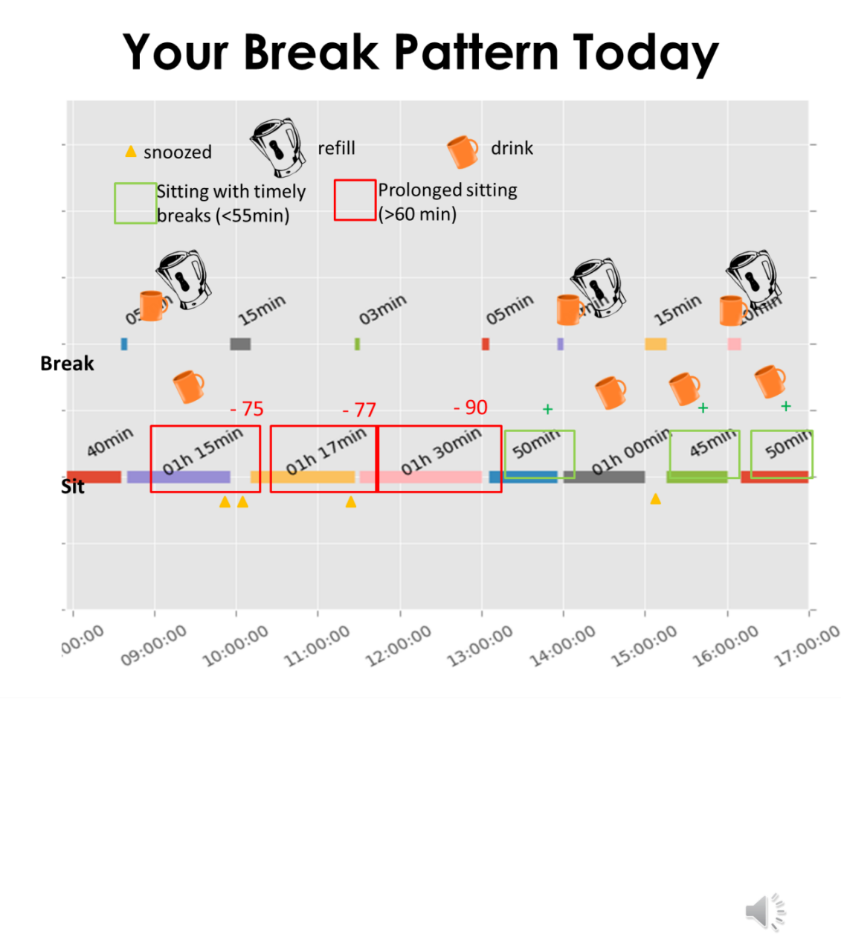


## Wireframes for the Android App
