# Supplementary material for: Using Internet of Things to Reduce Office Workers’ Sedentary Behavior: Intervention Development Applying the Behavior Change Wheel and Human-Centered Design Approach
Source: JMIR Mhealth Uhealth. 2020 Jul 29;8(7):e17914. doi: 10.2196/17914 (PMC7424484; doi:10.2196/17914)
Supplement: Multimedia Appendix 6 [file mhealth_v8i7e17914_app6.docx]

## Participant “cheat sheet”

### For baseline weeks


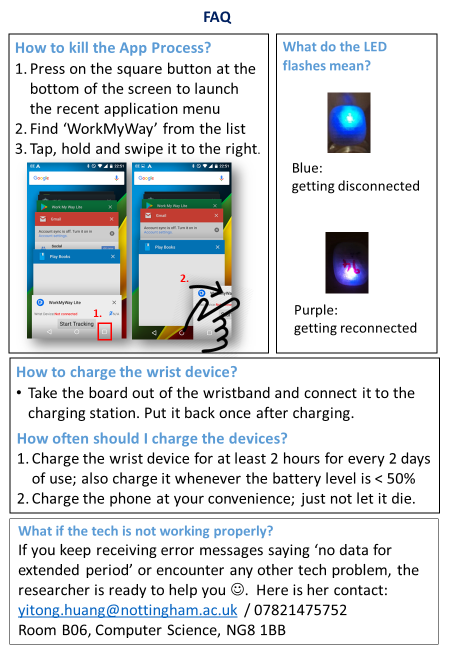

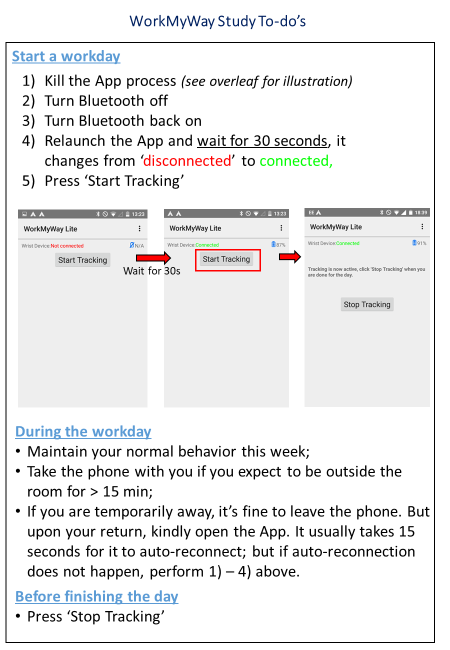


### For intervention period


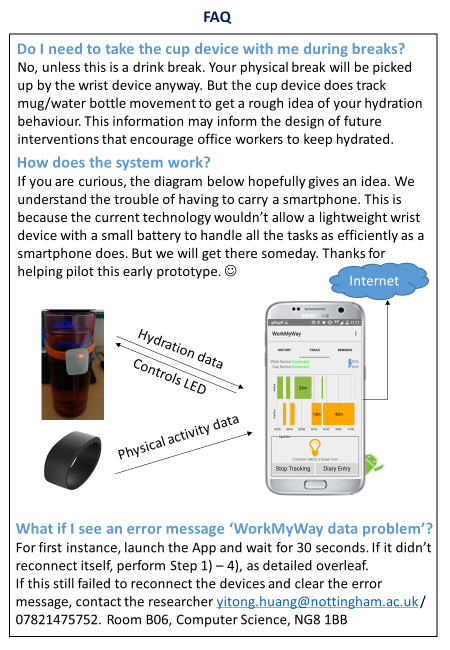

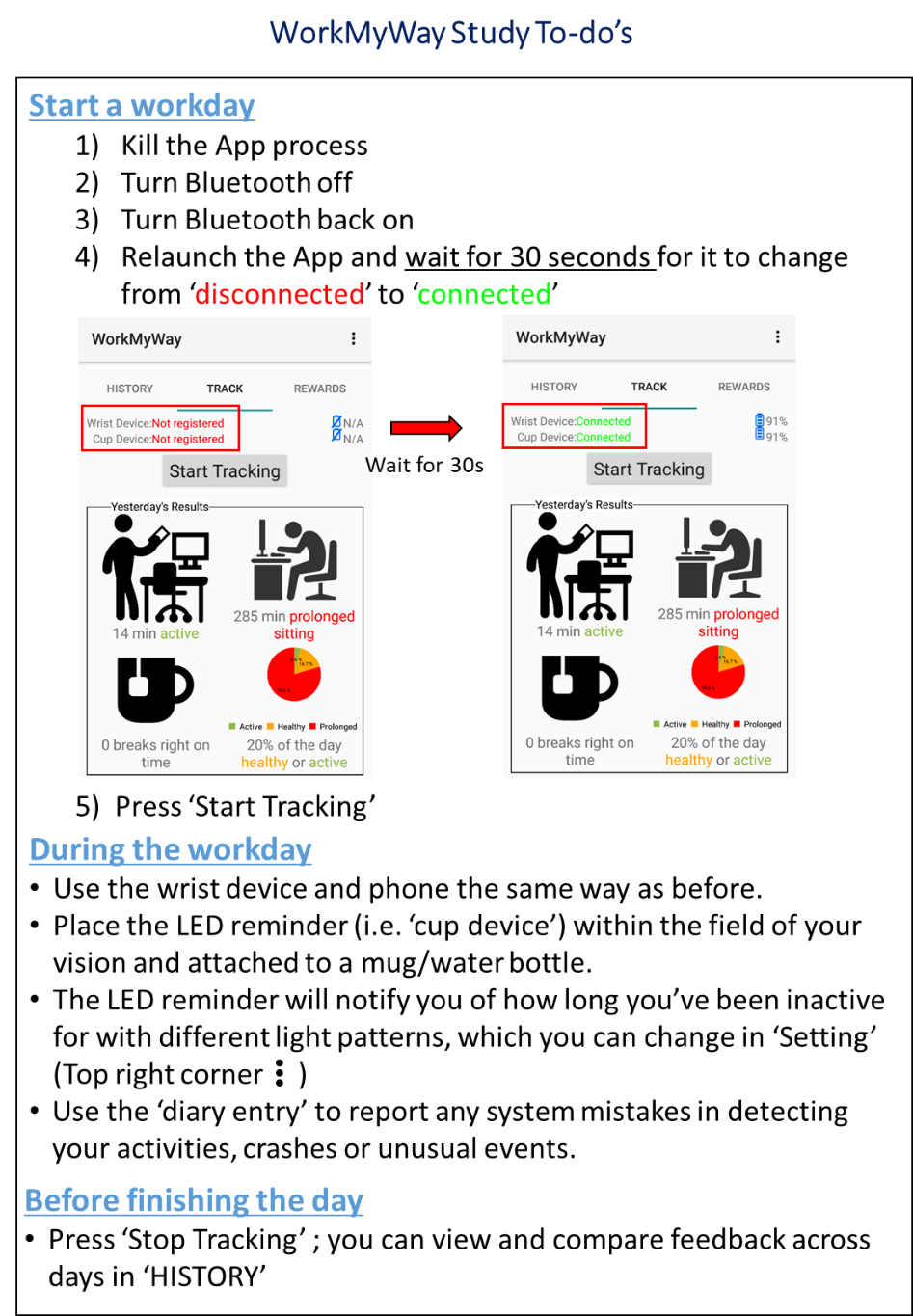


## Brief Action Planning protocol


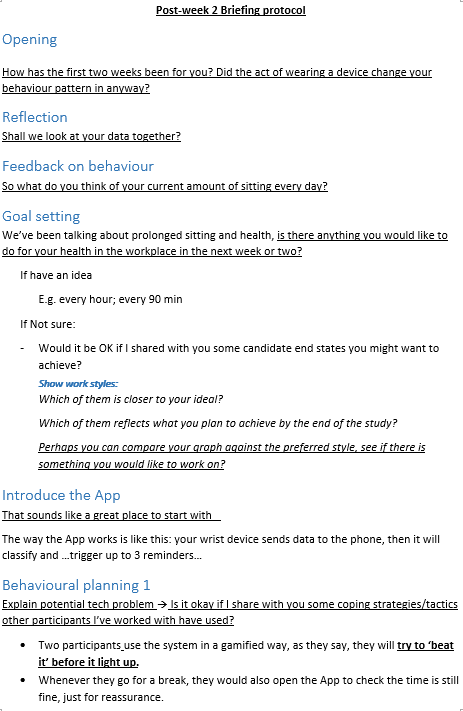


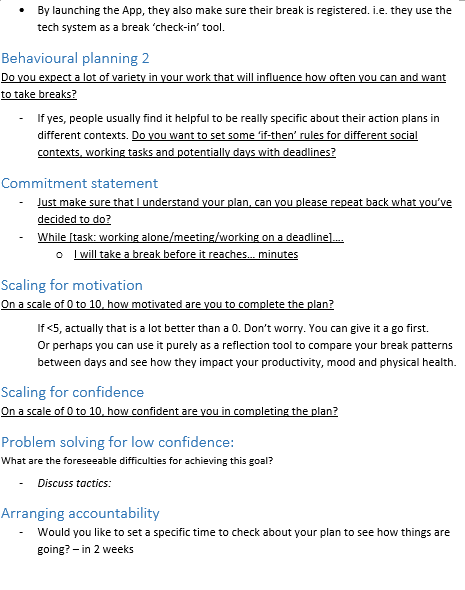


### Examples of baseline data vignette provided to participants at the action planning session


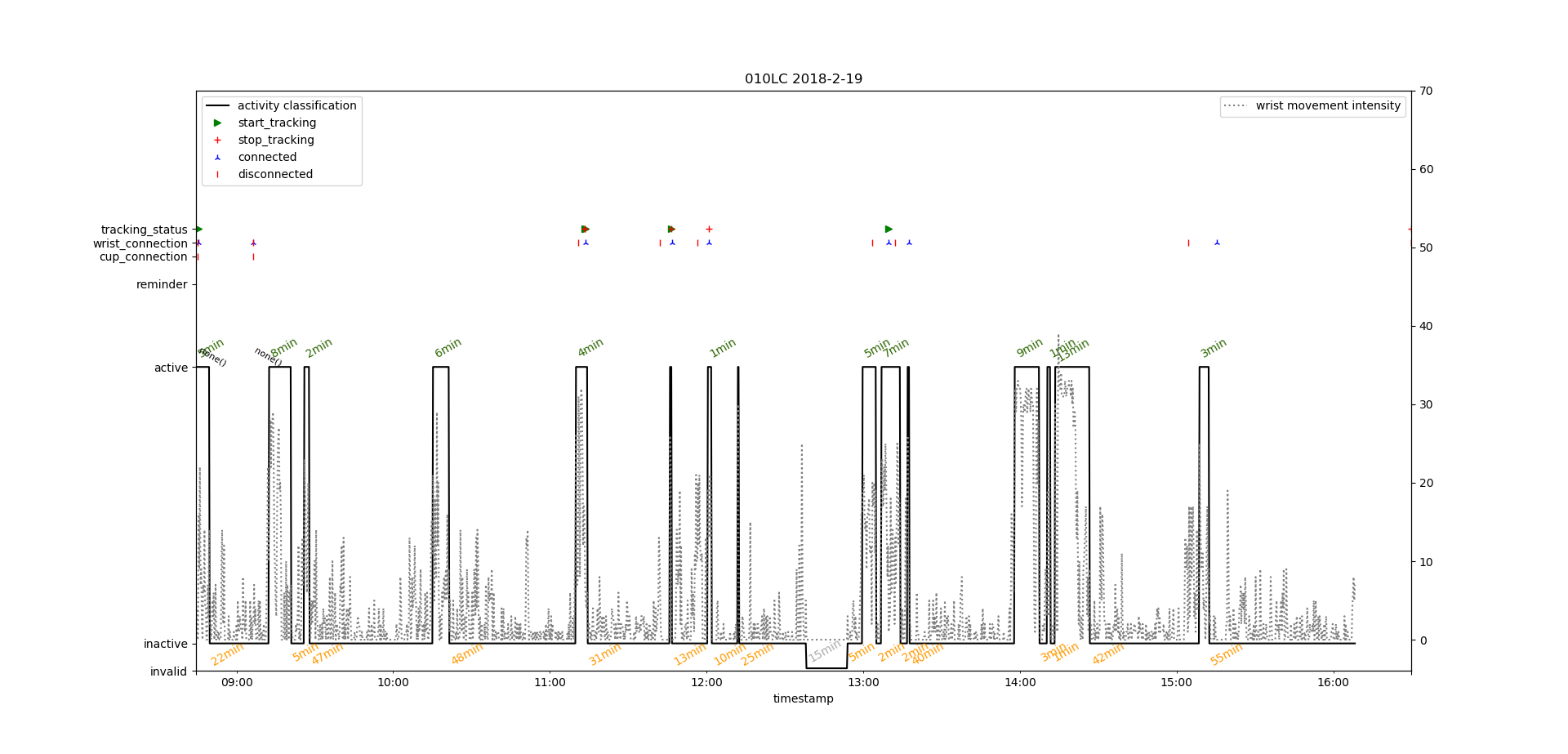


## Follow-up email in the 3^rd^ week during intervetnion period

Morning XXX,

It’s been 2 weeks since you started using the full *WorkMyWay* system. I’d like to remind you to review the ‘history’ section in the App and compare your performance against the original target you set for yourself two weeks ago (i.e. take a break every hour). You can change the reminder frequency yourself in the ‘setting’ menu on the top right corner.

If you think the device is too sensitive or not sensitive enough in detecting your breaks, please also let me know. I can help adjust the thresholds for you to suit your work contexts.

Finally, any feedback on the system would be welcomed. If you want to chat about your progress and provide interim feedback on the study and technology, we can book an informal meeting. This is optional and entirely up to you.

Best Regards,

[Researcher name]
